# Impact of leucine-rich diet on placenta's proteomic and metabolomic profiles of pregnant tumor-bearing rats

**DOI:** 10.1080/15384047.2026.2670792

**Published:** 2026-05-14

**Authors:** Igor Fernando dos Santos, Carla de Moraes Salgado, Victor Corasolla Carregari, Rogerio Williams dos Santos, Daniel Martins de Souza, Laís Rosa Viana, Maria Cristina Cintra Gomes-Marcondes

**Affiliations:** a Laboratory of Nutrition and Cancer – Structural and Functional Biology Department, University of Campinas, Campinas, São Paulo, Brazil; b Laboratory of Neuroproteomics, Biochemistry and Tissue Biology Department, University of Campinas, Campinas, São Paulo, Brazil; c D'Or Institute for Research and Education (IDOR), São Paulo, Brazil; d Instituto Nacional de Biomarcadores Em Neuropsiquiatria, Conselho Nacional de Desenvolvimento Científico e Tecnológico, São Paulo, Brazil; e INCT in Modelling Human Complex Diseases With 3D Platforms (Model3D), Conselho Nacional de Desenvolvimento Científico e Tecnológico, São Paulo, Brazil

**Keywords:** Cancer, nutritional supplementation, pregnancy, placenta, proteomic and metabolomic profile

## Abstract

**Background:**

Cancer during pregnancy is a fragile scenario where the maternal body undergoes significant molecular changes, including at proteomic and metabolomic levels, with damage to placenta and fetal development. Proteomic and metabolomic interaction analyses can clarify the complex dynamics in cancer associated with pregnancy. Leucine is involved in protein synthesis and metabolic regulation and may attenuate tumor-induced damage.

**Objective:**

We investigated placental proteomic and metabolomic profiles in the complex relationships among pregnancy, cancer, and leucine supplementation.

**Materials:**

Exploratory, non-targeted proteomic (mass spectrometry) and metabolomic (NMR spectrometry) analyses were performed on the placenta tissue of four pregnant groups: control (PC), Walker-256 tumor-bearing (PW), leucine (PL), and tumor-bearing fed a leucine-rich diet (PWL). Statistical comparisons were performed on technical replicate data within each group.

**Results:**

Tumor affected maternal weight, but leucine attenuated the decrease in placental weight and fetal resorption. Leucine supplementation restored the placental proteins involved in the nuclear structure, cytoskeleton, and immune response. Tumor evolution impaired pathways related to protein folding and chromosome localization, whereas leucine modulated interferon-gamma and hydrogen peroxide responses. PW placentas exhibited decreased adenosine, glucose, glutamate, and succinate, with enhanced lactate, creatine, and histamine levels, whereas leucine supplementation restored adenosine and pyridoxine and reduced lactate levels. The affected alanine/aspartate/glutamate and purine metabolism, beta-alanine metabolism, and the citrate cycle were minimized in leucine treatment (PWL), which mainly modulates purine and alanine/aspartate/glutamate pathways and pyruvate metabolism.

**Conclusion:**

A leucine-rich diet partially restored placental protein and metabolite levels, leading affected pathways to shift towards amino acid biosynthesis and pyruvate metabolism and minimizing effects on fetal development.

## Introduction

Cancer during pregnancy is a rare condition (1 in each 1000 pregnancies) that occurs when cancer develops during pregnancy or within 1-y postpartum.^1^^,^^2^ Despite being rare, pregnancy during breast cancer incidence has been rising in recent years due to lifestyle changes, such as postponing pregnancy.^1-3^ Given the rare occurrence of these two processes, the development of cachexia could happen with very low probability in these patients; therefore, there is a significant risk of underestimation, especially when considering the most common cancers diagnosed during pregnancy, such as breast and cervical cancers.^4^^,^^5^ As a complex process, pregnancy involves profound maternal adaptations that lead to maternal metabolic and hormonal adjustments to support the healthy development of the fetus.^6^ Those maternal adaptations are managed by the placenta, which acts as a fundamental selective barrier and communication interface.^6^

The interaction between gestation and cancer emerges as a complex challenge that compromises both fetal and maternal health, generating consequences that may include gestational loss, fetal growth restriction, low birth weight, and pathologies that can extend throughout the offspring's lifetime.^7^^,^^8^ Given the essential role of placental functions, these consequences probably stem from impaired placental development and activity caused by molecular changes in response to influence from tumoral development. The molecular consequences of cancer in placental tissue may include alterations in metabolic profiles^8^ and imbalance of protein synthesis and degradation pathways, although, to our knowledge, the changes in placental proteins and metabolites in this context have not been investigated. Since proteomics analysis can contribute to a better understanding of disease-related perturbations,^9^ and the metabolomic profile can predict some direct consequences,^10^ the integrated use of high-throughput multi-omics techniques allows the analysis of complex proteomic and metabolomic profiles, especially in the context of cancer during pregnancy.^11^

Given the harmful effects of cancer during pregnancy, including host metabolic and protein alterations, nutritional interventions emerge as a promising therapeutic approach that could provide maternal and fetal benefits.^12-15^ Among these, coadjutant treatment, leucine, an essential amino acid known for stimulating protein synthesis through the activation of the mTOR pathway,^16^ can act as a potential nutritional supplementation to mitigate the decreased protein synthesis and minimize protein wasting caused by cancer development, as seen in our previous studies in muscle tissues.^13^^,^^17-20^ Consistent with our findings and the broader literature, leucine supplementation is safe and effective during pregnancy, producing gestational outcomes comparable to those of the control group, with no adverse effects on maternal and fetal weights, litter size, food intake, or maternal blood biomarkers.^13-15^^,^^21^ Considering that the female Wistar rat model has a great biological similarity to the human pregnancy and that, to our knowledge, no alternative cancer model is currently suitable for experimental pregnancy studies, therefore, based on our previous publications, the Walker-256 carcinosarcoma is more suitable and matches the pregnancy development (20-d gestation period), despite being an experimental cancer cachexia model. Since there is still a lack of comparative multi-omics studies of placental tissues, including those related to nutritional supplementation, across both clinical and preclinical designs, particularly in relation to cancer, our study aims to characterize the changes in placental proteomic and metabolomic profiles in pregnant Walker-256 tumor-bearing rats and assess whether leucine-rich dietary supplementation can help reduce or alleviate these potential molecular disruptions.

## Materials and methods

### Study design

**Figure 1.**
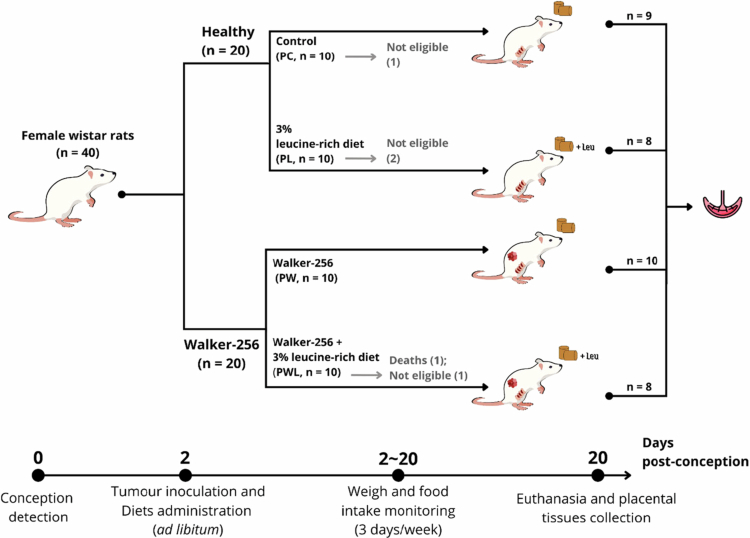
Experimental design of pregnant Wistar tumor-bearing rats, subject or not to nutritional supplementation with leucine. The timeline illustrates the procedures performed in pregnant rats, including pregnancy detection, Walker-256 tumor cell inoculation, dietary interventions, and sample collection. The tumor-bearing groups (PW and PWL) received 5 × 10^6^ Walker-256 cells via a right subcutaneous inoculation (as in previous studies.^6^^,^^11^^,^^15^) Animals were fed either a semi-purified normoproteic control diet (PC and PW) or a normoproteic 3% leucine-enriched diet (PL and PWL), according to AIN93^20^ *ad libitum*. Monitoring of body weight, food intake, and tumor evolution was measured three times per week. Euthanasia and sample collection were performed on the 20th day of pregnancy. The general UKCCCR guidelines for animal welfare^21^ and the ARRIVE guidelines 2.0^22^ were followed with ethics committee approval (CEEA/IB/UNICAMP, protocol # 6136-1/2022). Legend: PC—control group; PW—tumor-bearing group; PL—leucine group; and PWL leucine tumor-bearing group.

Forty adult virgin female albino Wistar rats (90 d old, 220–270 g body weight) were obtained from the Animal Facility Center (CEMIB at UNICAMP) and maintained in the Research Nutrition and Cancer Laboratory of the Department of Structural and Functional Biology at UNICAMP. The study was conducted in two consecutive experiments with 20 animals each. All the rats were housed in individual cages after mating overnight with males of the same strain at a ratio of one male:two females, following the harem method described by Baker.^22^ The first day of pregnancy was defined as when a vaginal smear detected the presence of spermatozoa. When pregnancy was confirmed, approximately 5 × 10^6^ Walker-256 viable cells were injected into the right subcutaneous flank of the tumor-bearing groups, and the semi-purified diet administration was started. The pregnant rats were given food and water *ad libitum* under light‒dark cycles (12/12 hours each), constant temperature (22 ± 2 °C), and humidity (50%–60%). The animals were monitored daily, and their weight and food intake were measured three times per week. Animal welfare was monitored weekly by facility veterinarians to minimize unnecessary suffering, and humane endpoints were defined as tumor necrosis, aggressive metastatic spread, and severe clinical symptoms, including a marked reduction in food and water consumption, limb and ocular pallor, and behavioral signs of distress. The experimental design is presented in Figure 1. The pregnant rats were randomly distributed into four experimental groups, with a minimum number of eight animals each, to guarantee at least minimal statistical confidence: two groups receiving a control diet (PC [*n* = 9], control group and PW [*n* = 10], Walker-256 tumor-bearing group), and two other groups fed with a leucine-rich diet (PL [*n* = 8], leucine control group and PWL [*n* = 8], leucine Walker-256 tumor-bearing group). Both semi-purified diets, control (C) and leucine-rich (L) diets, were prepared in accordance with the American Institute of Nutrition (AIN-93).^23^ Both diets had similar nitrogen levels (approximately 2.84 g N2/100 g diet), the same protein content of 18%, and were isocaloric, as in our previous research.^13^ The control diet (groups PC and PW) contained 18% protein (as 20% casein), 62.9% carbohydrate (39.7% corn starch, 13.2% dextrin, and 10% sugar), and 7% lipid and was completed with fiber and micronutrients. The leucine-rich diet (PL and PWL) differs only in carbohydrate concentrations (38.7% corn starch, 12.2% dextrin, and 9% sugar) and the addition of 3% leucine.^15^ Considering our previous works and the literature,^13-15^^,^^21^^,^^24-30^ the 3.0% L-leucine enrichment provided benefits and security in the health of the animals compared to those fed a normoprotein diet (AIN-93, as previously published in our work. After 20 d of pregnancy and tumor growth, all the animals were euthanized by decapitation after being anesthetized with 4% isoflurane, and their placentas were collected, weighed, and stored immediately at −80 °C for further analyses. Morphometric results were generated based on maternal body weight, placental and fetal weights, and the number of placentas, fetuses, and resorptions. *A priori*, pregnant rats' handling and exclusion criteria included animals with parturition occurring prior to the 20th gestational day (PC [*n* = 1], PL [*n* = 2], PW [*n* = 0], PWL [*n* = 2]). The general UKCCCR guidelines for animal welfare^31^ and the ARRIVE guidelines 2.0^32^ were followed, and the experimental protocols were approved by the Institutional Committee for Ethics in Animal Research (CEEA/IB/UNICAMP, protocol # 6136-1/2022).

The outcomes analyzed in this study included morphometric states, label-free protein abundance, metabolite content, protein‒protein interaction network, and biological pathways related to both proteomic and metabolomic results. The primary outcomes used to determine the sample size were exploratory proteomic and metabolomic analyses.

### Proteomic analysis via liquid chromatography-mass spectrometry

#### Sample extraction

The frozen placenta tissue samples for proteomic analysis were placed into a lysis buffer (100 mM Tris-HCl, pH 8, 150 mM NaCl, 1 mM EDTA, 0.5% Triton) containing protease inhibitors and homogenized according to protocols described by Davis-Anderson^33^ and Bourdin-Pintueles.^34^ Cellular lysates were subjected to successive washes and centrifugation to remove detergent, followed by protein digestion using trypsin via the FASP protocol.^35^ Briefly described here,^36^ each sample was processed using a YM-10 filter unit (Millipore, Carrigtwohill, Ireland) to concentrate proteins. 8 M urea in 0.1 M Tris/HCl, pH 8.5, was added, and the mixture was centrifuged twice at 14,000 × *g* at 20 °C for 15 minutes each. The residual material in the tube was then discarded. Subsequently, 8 mM DTT diluted in urea buffer was added (100 µl per FASP filter unit), and the mixture was incubated at 56 °C for 15 minutes. Following this, 0.05 M iodoacetamide diluted in 8 M urea was added. The samples were then incubated in the dark without agitation for 20 minutes. Three other centrifugations at 14,000 × *g* for 10 minutes were subsequently performed, after which the pellet was discarded. Then, 50 mM Ambic was added to the filter containing the protein extract, and then centrifuged three times at 14,000 × *g* for 10 minutes. Next, trypsin (Promega, Madison, WI) was added at a 1:100 ratio, and the material was vortexed for 1 minute. The samples were then incubated overnight at 37 °C. The filter was transferred to a new collection tube, and 50 mM Ambic was added, followed by two additional centrifugations. The filtered material containing the digested proteins was acidified with formic acid to a final concentration of 1% of the total volume to block trypsin activity.

#### Proteomics analysis (LC-MS) using Data-Independent Acquisition (DIA)

The separation of tryptic peptides was performed on an ACQUITY MClass System (Waters Corporation). A total of 1 μg of each digested sample was loaded onto a Symmetry C18 5 μm, 180 μm × 20 mm precolumn (Waters Corp.) used as a trapping column and subsequently separated by a 120 minutes reversed-phase gradient at 300 nL/minute (linear gradient, 3%–55% ACN over 90 min) using an HSS T3 C18 1.8 μm, 75 μm × 150 mm nanoscale and LC column (Waters Corp.) maintained at 30 °C. For gradient elution, water–formic acid (99.9/0.1, v/v) was used as eluent A, and acetonitrile–formic acid (99.9/0.1, v/v) was used as eluent B. The separated peptides were analyzed by high-definition Synapt G2-Si mass spectrometer directly coupled to the chromatographic system. Differential protein expression was evaluated with a data-independent acquisition (DIA) of shotgun proteomics analysis by expression configuration mode (MSe). The mass spectrometer was operated in “Expression Mode” and switched between low (4 eV) and high (25–60 eV) collision energies on the gas cell, using a scan time of 1.0 s per function over 50–2000 m/z. All the spectra were acquired in ion mobility mode by applying a wave velocity for ion separation of 1.000 m/s and a transfer wave velocity of 175 m/s. The processing of low and elevated energy, added to the data of the reference lock mass ([Glu1]-Fibrinopeptide B Standard, Waters Corp.), provides a time-aligned inventory of accurate mass retention time components for both the low and elevated-energy (EMRT, exact mass retention time), each sample was run in three technical replicates. Continuum LC‒MS data from three replicate experiments for each sample were processed for qualitative and quantitative analysis using the software Progenesis (Waters Corp.). The processed samples, as described above, underwent liquid chromatography coupled with a high-definition mass spectrometer (Synapt G2-Si Mass Spectrometry, Q-ToF, Waters, Miami, USA) and were analyzed using high-definition MSE mode. The search parameters were set for automatic tolerance of precursor and fragment ions (10 ppm), a minimum of three combined fragments per peptide, a minimum of three paired fragments per protein, a minimum of two combined peptides per protein, two trypsin cleavages, carbamidomethylation of cysteines, and oxidation of methionines as fixed and variable modifications. The false discovery rate (FDR) for identification was set below 1%. For the search for phosphorylated peptides, variable phosphorylation modifications on STY (serine, threonine, and tyrosine) were included.

#### Peptide quantification and protein abundance (Label-Free)

Each sample was analyzed in triplicate with technical replicates. The analysis of differentially expressed proteins followed the methods proposed by Silva^37^ and Visser.^38^ The continuous LC‒MS data from three technical replicates for each sample were processed for qualitative and quantitative analysis using Progenesis QI for proteomics software (Waters Corp.).

### Metabolomic analysis via nuclear magnetic resonance

#### Sample extraction

Approximately 0.3 g of placental tissue was homogenized in a solution containing methanol and chloroform (2:1, v/v) and sonicated (VCX 500, Vibra-Cell; Sonics & Materials Inc., Newtown, CT, USA) for 3 minutes on ice, following the protocol proposed by Le Belle.^39^ After homogenization, a cold solution with Milli-Q water and chloroform (2:1, v/v) was added to each sample. The samples were briefly vortexed and left to rest for 15 minutes. They were then centrifuged at 3000 × *g* for 20 minutes at 4 °C. After centrifugation, the polar phase was collected and lyophilized using a vacuum concentrator (Vacufuge Concentrator; Eppendorf, Hamburg, Germany). For ¹H-NMR spectra acquisition, each lyophilized sample was resuspended in D₂O, phosphate buffer (0.1 M, pH 7.4), and TMSP-d₄.

#### Metabolomic analysis using ¹H-NMR spectra

The resulting solution of placenta extraction was analyzed to obtain ¹H-NMR spectra, using a Varian Inova NMR spectrometer (Agilent Technologies Inc., Santa Clara, CA, USA), a ¹H resonance frequency of 500 MHz at a constant temperature. A total of 256 free induction decays were recorded, with 32 K data points spanning a spectral width of 16 ppm, ensuring optimal signal acquisition. A 1.5 s relaxation delay was implemented between scans, during which a continuous radiofrequency field was applied for water pre-saturation.

#### Metabolites identification and quantification

Following data collection, spectral preprocessing was conducted by including Fourier transformation, phasing, and baseline corrections, deletion of the water region, shim correction, apodization (line broadening with lb ~0.3), and reference adjustment. Metabolite identification and quantification were carried out using Chenomx RMN Suite software (Chenomx Inc. version 12.02, Edmonton, Canada) through computer-assisted manual fitting. The samples were analyzed in a blinded manner to minimize bias, with the same evaluator fitting the identified metabolites to each spectrum. There were no relevant ambiguities in the assignments performed in this study; therefore, the use of 1D NMR spectra processed through Chenomx was technically appropriate and sufficient for the reliable identification of metabolites in placenta samples analyzed in this work. This methodology was based on the literature and our previous results.^8^^,^^40-45^ The final metabolite profiles were normalized to tissue weight, and concentrations were expressed as millimoles per milligram of tissue.

### Statistical analyses

All the statistical analyses followed the normality test of Shapiro–Wilk (GraphPad Prism software, version 9.5.1.733, San Diego, CA, USA) to guarantee the use of appropriate statistical approaches. When the data did not meet the normality criteria, normalization was performed.

For multiple group comparisons, the morphometric data were analyzed using a two-way ANOVA followed by the Tukey post hoc test (GraphPad Prism software, version 9.5.1.733, San Diego, CA, USA), comparing maternal body weight, body weight gain, number of placentas and fetuses, and number of resorptions between the groups. The results are expressed as the mean and standard deviation (SD) and were considered significant when the *p*-value was ≤0.05.

Proteomic expression analysis was conducted considering available technical replicates for each experimental condition, assuming each group was an independent variable. Protein identification was based on the detection of more than two fragmented ions per peptide and at least two peptides per protein. The list of normalized proteins was tracked based on the following criteria: a protein identified in at least 2 out of 3 runs of the same sample (2 technical replicates) with a regulation change exceeding ±20%; only modulated proteins were considered significant when the *p*-value was ≤0.05. The proteomic data obtained were compared across the four experimental groups to identify changes in protein expression. The total number of proteins identified was 1062, and the altered proteomic profile is presented in Supplementary Table 1. The aim was to map potential mechanisms of upregulated or downregulated regulation, as well as proteins exclusively found in specific groups.

The results were applied to identify protein‒protein interaction (PPI) networks using String Database (version 12.0) alongside Cytoscape (version 3.10.3). From the PPI graphs, the Gene Ontology (GO) database was used to establish the main biological process pathways altered in each comparison. Pathways were ranked according to their signal value (weighted harmonic mean balancing significance (−log(FDR)) and enrichment (observed/expected ratio)), false discovery rate (FDR) values, and sizes representing raw numbers of altered proteins. The three most altered biological processes from each comparison were further analyzed via PPI to understand the main proteins involved and their direction of modulation (downregulation or upregulation).

All the ¹H-NMR data analyses were performed using the online platform MetaboAnalyst 6.0 (a statistical, functional, and integrative analysis of metabolomic data), showing the discrepancies between the identified metabolites and their concentrations among the experimental groups.^46^ A total of 54 metabolites were identified across all the placental tissue samples and are available in Supplementary Table 2. An integrated analysis was performed from metabolomic and proteomic profiles to identify the impacted pathway in placental tissue using the MetaboloAnalyst platform (MetaboAnalyst 6.0. https://www.metaboanalyst.ca/MetaboAnalyst/).^46^ The principal component analysis (PCA) was implemented, as well as the partial least squares discriminant analysis (PLS-DA) models, and the data were extracted at a confidence level of 95%.

## Results

### Tumor evolution impaired morphometric parameters in pregnant rats

Consistent with our previous findings, tumor development negatively affected maternal and fetal morphometric parameters in both tumor-bearing groups (PW and PWL).^13^^,^^19^^,^^20^ Maternal final body weight was reduced in PW by approximately 15% in comparison to the control groups (PC and PL), while body weight gain decreased by 44% and 49% in PW and PWL groups, respectively. The tumor weight represented approximately 18% of the initial body weight in both tumor-bearing groups. The initial and final food intakes were similar in the leucine-pregnant group compared with the control group. Although, as a damaging effect of the tumor, there was a significant decrease in food intake in both tumor-bearing groups, showing the anorexia process (Table 1). The leucine content in maternal serum was increased in both leucine-rich diet groups (data not shown), assuring the effect of nutritional supplementation. Placental weight decreased significantly only in the PW group, while fetal weight decreased in both tumor-bearing groups; however, the rate of decrease was lower in PWL than in the PW group. The fetal/placental weight ratio was reduced in both tumor-bearing groups (PW = 2.33 and PWL = 3.77) compared to control groups, reflecting a higher incidence of fetal resorption. However, the fetal resorption rate was attenuated in the leucine-supplemented group (Table 1).

**Table 1.** Morphometric profiles of *Wistar* pregnant *Walker*-256 tumor-bearing rats, subjected or not to 3% leucine-rich diet and compared to control groups.

Morphometric parameters	Groups			
	PC	PL	PW	PWL
Initial body weight (g)	225.88 ± 22.93	227.10 ± 17.86	216.23 ± 17.69	223.07 ± 15.51
Final body weight (g)	315.78 ± 24.73	316.13 ± 29.32	266.15 ± 17.70*	268.30 ± 47.43**
Body weight gain (g)	89.89 ± 25.52	89.03 ± 23.19	49.92 ± 22.75*	45.22 ± 36.44**
Initial food intake (g/d)	15.33 ± 0.58	14.80 ± 0.84	15.17 ± 0.75	15.33 ± 0.82
Final food intake (g/d)	29.67 ± 1.53	29.80 ± 0.45	26.50 ± 1.05*	27.17 ± 0.98
Number of placentas	10.89 ± 2.26	11.00 ± 1.19	9.40 ± 2.87	10.00 ± 2.61
Placental weight (g)	0.44 ± 0.03	0.42 ± 0.05	0.30 ± 0.11*	0.41 ± 0.15
Fetuses weight (g)	2.85 ± 0.81	3.50 ± 0.22	0.70 ± 0.94*	1.53 ± 1.70**
Number of reabsorptions	0.44 ± 0.52	0.13 ± 0.35	1.30 ± 1.05	0.86 ± 1.06

Body weight gain corresponds to the difference between the final body weight and the body weight(g) at zero day. Legend: PC: control pregnant group; PL: pregnant rats fed a 3% leucine-rich diet; PW: pregnant Walker tumor-bearing group; PWL: tumor-bearing pregnant rats fed a 3% leucine-rich diet. Values are presented in means ± standard deviation (SD). *p < 0.05 difference against PC group; **p < 0.05 difference against PL group. Values obtained via two-way ANOVA analysis followed by Tukey's post-test, except for tumor weight, which was obtained via a non-paired *t*-test.

### The placental proteomic profile was significantly altered in pregnant tumor-bearing rats, but the leucine-supplemented group exhibited a minimized alteration

**Figure 2.**
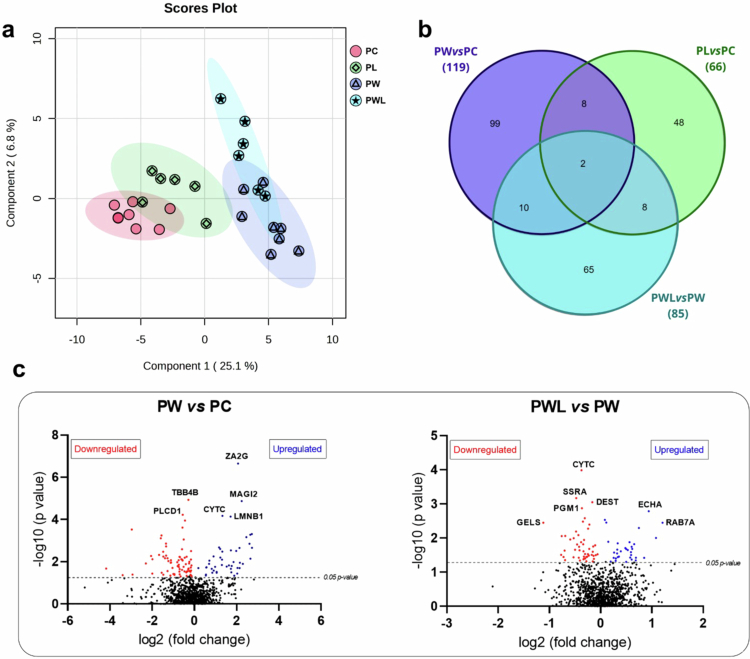
Proteomic profile of placental tissue from different experimental groups, implanted with Walker-256 tumor cells and subjected to or not to leucine-rich diet. (a) Partial least squares discriminant analysis (PLSDA) plot illustrating groups. The experimental groups are represented by colored clusters (red = PC (Control), green = PL (Leucine), dark blue = PW (Walker-256), and light blue = PWL (Walker-256 + Leucine). There was a clear segregation between groups, with some overlap between PW and PWL groups. This is particularly relevant, as leucine supplementation is a dietary intervention with systemic effects that are inherently less pronounced than those of pathological processes such as tumor development. (b) Venn diagram displaying differentially expressed proteins unique to each comparison (PW *vs* PC: 99; PL *vs* PC: 66; PWL *vs* PW: 65) and shared between comparisons (PW *vs* PC ∩ PL *vs* PC: 8; PW *vs* PC ∩ PWL *vs* PW: 10; PL *vs* PC ∩ PWL *vs* PW: 8; all three: 2). (c) Volcano plots illustrating differentially expressed proteins between the comparison of the PW *vs* PC groups (left graphic) and between the PWL *vs* PW groups (right graphic). Fold-change values were converted to a base 2 logarithm, and *p*-values to a base -10 logarithm. The horizontal line indicates the significance threshold (*p*-value in −log10 ≥ 2), distinguishing significantly altered proteins from non-significant ones. Proteins with decreased expression are displayed on the left, while those with increased expression are shown on the right. Legend: PC (Control), PL (Leucine), PW (Walker-256), and PWL (Walker-256 + Leucine); ZA2G (Zinc-alpha-2-glycoprotein), TBB4B (Tubulin beta-4B chain), MAGI2 (Membrane-associated guanylate kinase, WW and PDZ domain-containing protein 2), PLCD1 (1-phosphatidylinositol 4,5-bisphosphate phosphodiesterase delta-1), CYTC (Cystatin-C), LMNB1 (Lamin-B1), SSRA (Translocon-associated protein subunit alpha), PGM1 (Phosphoglucomutase-1), GELS (Gelsolin), DEST (Destrin), ECHA (Trifunctional enzyme subunit alpha, mitochondrial), and RAB7A (Ras-related protein Rab-7a).

The profiles on the principal component analysis plot showed a visible separation among the groups (Figure 2a), with some overlap between healthy groups (PC and PL) and tumor-bearing groups (PW and PWL), which is expected considering that leucine supplementation promotes some adaptation but does not completely change the placental profile. Among the 1062 identified proteins, 146 presented significant modulations. The PW group had the largest number of altered proteins when compared to PC (119 proteins), followed by PWL compared to PW (85 proteins) and finally PL group compared to PC group (66 proteins). The total number of altered proteins in each comparison: PW *vs* PC, PL *vs* PC, and PWL *vs* PW, is presented in Figure 2b. Notably, 99, 65, and 48 proteins were exclusively modulated in the PW, PWL, and PL groups, respectively, indicating distinct proteomic signatures for each condition. Additionally, there were protein overlaps among the comparisons: 12 proteins were common to both PW *vs* PC and PWL *vs* PW, and 10 proteins were shared between PWL *vs* PW and PL *vs* PC, while only 2 proteins were altered across all three comparisons (Figure 2b).

Placental tissue underwent damage due to tumor evolution, as illustrated in Figure 2c and Supplementary Figure 1, which show the main proteins altered among the groups and the direction of these alterations. The PW *vs* PC comparison exhibited a notable upregulation of proteins, including zinc-alpha-2-glycoprotein (ZA2G; Q63678) and membrane-associated guanylate kinase inverted 2 (MAGI2; O88382), alongside a downregulation of tubulin beta-4B chain (TBB4B; Q6P9T8). In addition, the main effect of leucine supplementation in placental tissue, observed when PL was compared to PC (Supplementary Figure 1), showed upregulation of inositol polyphosphate 5-phosphatase OCRL (OCRL; D3ZGS3), which counteracted the downregulated proteins lactadherin (MFGM; P70490) and serpine 1 (PAI1; P20961). Regarding the association between leucine supplementation and tumor evolution (PWL *vs* PW), we observed downregulation of Destrin (DEST; Q7M0E3), translocon-associated protein subunit alpha (SSRA; Q7TPJ0), and Cystatin-C (CYTC; P14841).

**Figure 3.**
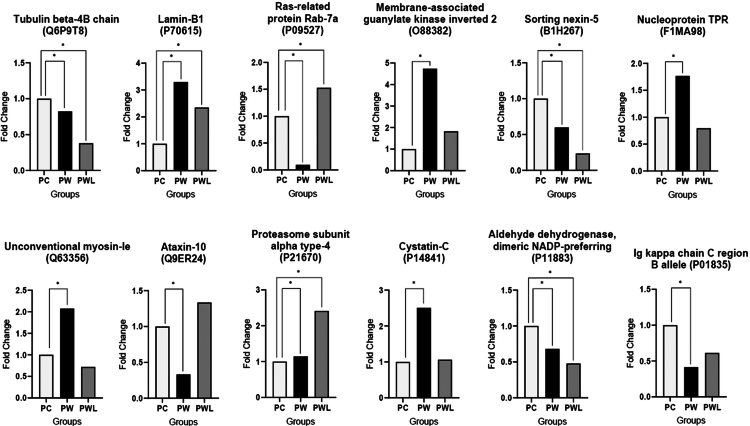
Shared altered proteins between PWL and PW, when compared to the control group (PC). To better understand the effects of leucine supplementation, the 12 proteins (identified by at least 2 unique peptides) altered in both the PW *vs* PC and PWL *vs* PW comparisons were analyzed using fold change (FC) values. PW and PWL fold changes were normalized against PC fold change, here stated as “1”. *p <  0.05 difference against PC group. Legend: PC (control), PW (Walker-256), and PWL (Walker-256 + Leucine).

Among the 12 proteins commonly altered in both the PW *vs* PC and PWL *vs* PW comparisons, we presented the analysis based on fold change (FC) values relative to those of the control group to better understand the effects of leucine supplementation (Figure 3). Treatment with leucine supplementation (PWL) brought the levels of these 8 proteins—membrane-associated guanylate kinase inverted 2 (O88382), Ig kappa chain C region B allele (P01835), Lamin-B1 (P70615), Ataxin-10 (Q9ER24), Ras-related protein Rab-7aC (P14841), Cystatin (P14841), Unconventional myosin-Ie (Q63356), and Nucleoprotein TPR (F1MA98)—closer to those of the control group (PC). Most of these proteins are related to nuclear and cytoskeletal structure, cell signaling, and the immune response. While the other four proteins (Tubulin beta-4B chain (Q6P9T8), Aldehyde dehydrogenase, dimeric NADP-preferring (P11883), Sorting nexin-5 (B1H267), and Proteasome subunit alpha type-4 (P21670)) showed values different from PC, indicating increased tumor-related effects independent of leucine supplementation.

### Protein networks and biological pathways impacted by tumor evolution and under leucine supplementation

Protein-protein interaction (PPI) networks from the altered proteins are presented in Supplementary Figure 2, revealing the main proteins that were upregulated or downregulated and directly interfering with biological pathways.

Placentas from the tumor-bearing group (PW) presented downregulation of several proteins, with the most intensely affected being ATP synthase subunit f, mitochondrial (Atp5mf) and Ras-related protein Rab-7a (Rab7a). Among the upregulated proteins, neurofilament heavy polypeptide (Nefh), nuclear migration protein nudC (Nudc), eukaryotic translation initiation factor 4 H (Eif4h), small ribosomal subunit protein eS7 (Rps7), and PAT complex subunit CCDC47 (Ccdc47) showed the highest levels of upregulation (Supplementary Figure 2a). In contrast, the leucine supplementation (PL; Supplementary Figure 2b) led to a different pattern, as the placental tissue presented a higher upregulation in synthesis of Stat3 and Stoml2, and downregulation of the nucleoprotein TPR (Tpr) and far upstream element-binding protein 2 (Khsrp). Regarding the leucine-treated tumor-bearing group (PWL; Supplementary Figure 2c), the placenta tissue presented higher downregulation of the protein gelsolin (Gsn), while Ras-related protein Rab-7a (Rab7a) was the most upregulated one, opposite to what was observed in the PW group.

**Figure 4.**
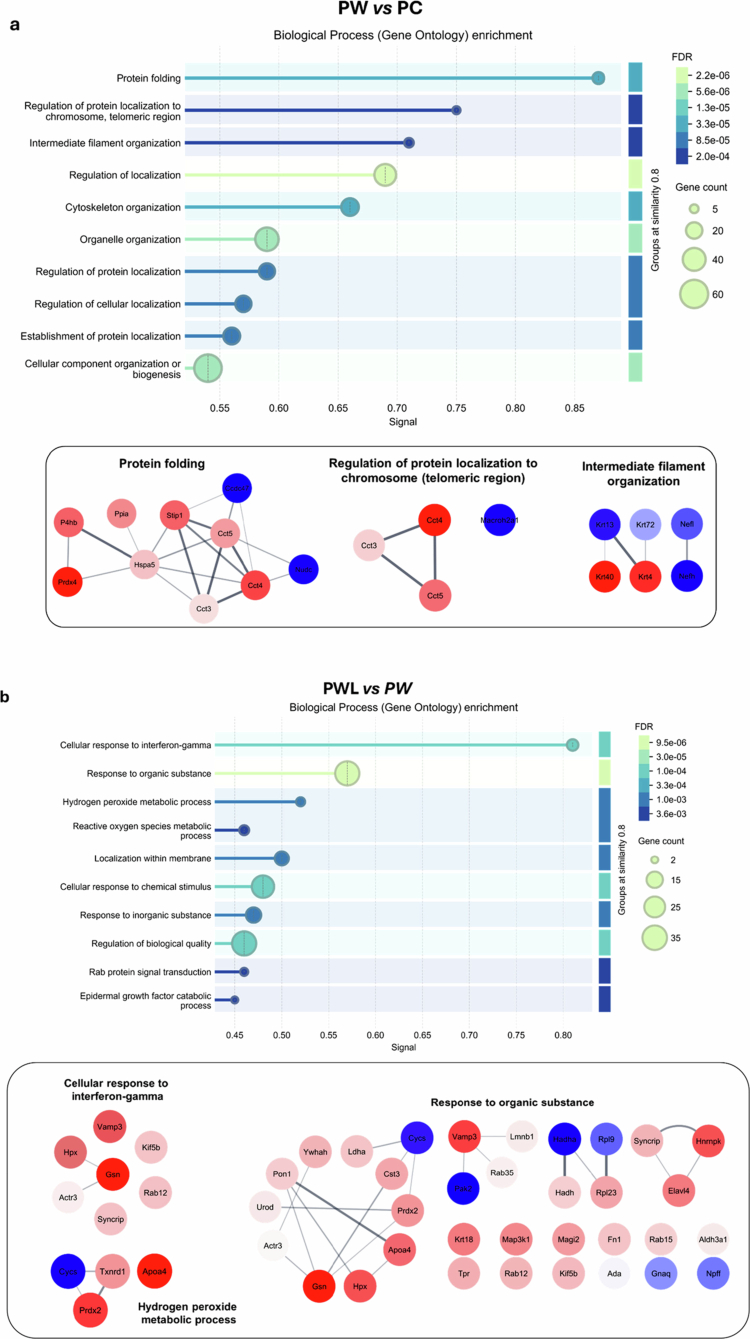
Protein networks and biological pathways were impacted in placental tissue from tumor-bearing rats subjected to or not to a leucine-rich diet. (a) Pathways with higher impact (Signal) (top) show the altered biological processes from the Gene Ontology Database pathways (GO) in the Walker-256 tumor-bearing group (PW) compared to the control (PC). Data derived from PPI networks generated in String. Pathways are ranked by pathway signal value; a weighted harmonic mean balancing significance (-log(FDR)) and enrichment (observed/expected ratio). The scale on the right displays false discovery rate (FDR) values, while the dot sizes represent the raw number of altered proteins. Protein interaction networks of the three GO pathways (bottom) with the highest signal values in the Walker-256 tumor-bearing group (PW) compared to the control group (PC). Node colors indicate the type of regulation (red: decreased synthesis; blue: increased synthesis), with color intensity reflecting FoldChange values for protein folding; Regulation of protein localization to the chromosome, telomeric region; and intermediate filament organization. (b) Pathways with higher impact (Signal) (top) show the altered biological processes from GO pathways in the leucine-treated Walker-256 tumor-bearing group (PWL) compared to the tumor-bearing group (PW). Protein interaction networks of the three GO pathways (bottom) with the highest signal values in the leucine-treated Walker-256 tumor-bearing group (PWL) compared to the tumor-bearing group (PW), with color intensity reflecting fold change values for the cellular response to interferon-gamma; the hydrogen peroxide metabolic process; and the response to organic substances.

The altered biological pathways obtained from the comparisons PW *vs* PC (Figure 4a), PL *vs* PC (Supplementary Figure 3), and PWL *vs* PW (Figure 4b) were followed by the protein networks from the three main pathways for each comparison. Data generated in String, along with data from the Gene Ontology (GO) database, were used. The significance of pathways was based on signal values (ratio FDR/gene count). The most significant pathways altered in the placentas from the PW group, in comparison to PC, were related to protein folding, regulation of protein localization to the chromosome, telomeric region, and intermediate filament organization. Here, the PPI networks presented mainly down-regulated proteins, such as peroxiredoxin-4 (Prdx4), T-complex protein 1 subunit delta (Cct4), and both keratin type II cytoskeletal 4 (Krt4) and keratin type I cytoskeletal 40 (Krt40), which are related to different cell activities, including cytoskeletal integrity and cell survival. The upregulated proteins, such as nuclear migration protein nudC (Nudc), core histone macro-H2A.1 (Macroh2a1), and neurofilament heavy polypeptide (Nefh), have a key role in maintaining cellular integrity and function, particularly in cell division and gene regulation. Most placental proteins of the PW group in these networks showed a significant reduction in synthesis, which could interfere with cell survival (Figure 4a).

The most significantly altered pathways in the placentas from the PL group, compared to PC, were the cellular response to heat, the response to estrogen, and intracellular protein transport (Supplementary Figure 3a). In the heat response pathway, heat shock proteins (Hsp90aa1, Hspd1, and Hsp90ab1) formed an upregulated cluster, while Tpr and Atp2a2 were downregulated as isolated nodes. The estrogen response showed upregulated Hsp90aa1 and Hspd1 clustering with downregulated Ldha, while C3 was upregulated and Mfge8 and Serpine1 were downregulated independently. For intracellular protein transport, a complex cluster included upregulated proteins (Hsp90aa1, Ywhah, Hspd1, Pmpca, Stat3) and downregulated ones (Gfap, Tomm34), suggesting antagonistic interactions. Several proteins (Copb1, Rab7a, Psap, Tpr, Sar1b) remained unclustered, with mixed regulation patterns in the PL group (Supplementary Figure 3b).

In the leucine-treated Walker-256 tumor-bearing group (PWL), compared to the tumor group fed a control diet (PW), the most significant pathways were related to the cellular response to interferon-gamma, the response to organic substances, and hydrogen peroxide metabolism. In the cellular response to interferon-gamma, all altered proteins were downregulated, particularly gelsolin (Gsn) and hemopexin (Hpx), which clustered together, while Vesicle-associated membrane protein 3 (Vamp3) appeared as an isolated node. The response to organic substances had the highest number of proteins that were significantly altered. Notably, Gsn was downregulated in the same cluster as cytochrome c, somatic (Cycs), which was upregulated. The serine/threonine-protein kinase PAK 2 (Pak2) was upregulated alongside Vamp3, which was downregulated, while the trifunctional enzyme subunit alpha, mitochondrial (Hadha), was also upregulated. In the hydrogen peroxide metabolic process, Cycs was upregulated in the same cluster where peroxiredoxin-2 (Prdx2) was downregulated. Additionally, apolipoprotein A-IV (Apoa4) was downregulated in the PWL group compared to PW group (Figure 4b).

### The metabolomics profile was affected by tumor evolution, and leucine supplementation minimized most of the alterations

The metabolomic profile analysis using partial least squares discriminant analysis (PLS-DA) (Supplementary Figure 4) showed experimental group segregation, though with less separation than observed in the proteomic data, with a slight overlap between the tumor groups, independent of nutritional supplementation.

The metabolites significantly altered in the placentas of the different groups are presented in Table 2, along with the following comparisons: PW *vs* PC, PWL *vs* PC, and PWL *vs* PW. When comparing the PW and PC groups, the main altered metabolites with decreased contents included adenosine (70% lower), aspartate, glucose, glutamate, and succinate (approximately 30%–40% lower), as well as glutathione and pyridoxine (approximately 60% lower). In contrast, there was an increase in creatine (1.9-fold), histamine (1.7-fold), and lactate (1.3-fold) levels. On the other hand, the comparison of PWL *vs* PW showed that leucine supplementation resulted in increased concentrations of adenosine and pyridoxine (approximately 2.0–3.0-fold) as well as other metabolites, including aspartate, asparagine, and leucine (around 1.7-fold), while lactate levels decreased by 23%. Interestingly, the tumor-bearing group supplemented with leucine (PWL) exhibited the most of those metabolites at similar levels to the PC group, except for histamine and asparagine (PWL *>* PC, which showed an opposite trend compared to PW *vs* PC).

**Table 2.** Metabolomic profile of *Wistar* pregnant *Walker*-256 tumor-bearing rats, subjected or not to 3% leucine-rich diet and compared to control groups.

					Tukey's HSD			
Metabolite	*F*-value	*p*-value	−Log10 (*p*)	FDR	PW *vs* PC	PL *vs* PC	PWL *vs* PW	PWL *vs* PC
Succinate	46.349	6.53E-10	9.1848	1.48E-08	↓	↑	—	—
Glucose	17.477	3.92E-06	5.4069	1.49E-05	↓	↓	—	↓
Glutamate	8.6501	0.00050248	3.2989	0.0014237	↓	—	↑	—
Aspartate	7.5132	0.0011195	2.951	0.0027188	↓	—	↑	—
Pantothenate	6.6704	0.0021033	2.6771	0.0047675	↓	—	—	↓
Adenosine	4.7527	0.010107	1.9954	0.018085	↓	—	↑	—
Creatine	17.687	3.57E-06	5.4477	1.49E-05	↑	—	↓	—
Lactate	8.2288	0.00067194	3.1727	0.0018277	↑	↑	↓	—
Histamine	4.2733	0.015464	1.8107	0.026289	↑	—	—	↑
Pyridoxine	2.5251	0.02822	1.5494	0.13853	↓	—	↑	—
Guanosine	65.231	2.12E-11	10.674	7.21E-10	—	↑	—	—
O-Phosphocholine	32.803	1.76E-08	7.7541	2.00E-07	—	↑	—	—
Inosine	27.137	9.81E-08	7.0084	6.63E-07	—	↑	—	—
Uracil	26.867	1.07E-07	6.9698	6.63E-07	—	↓	—	↓
Ethanolamine	25.714	1.58E-07	6.8022	8.94E-07	—	↓	—	↑
NAD+	23.527	3.40E-07	6.4679	1.78E-06	—	↑	—	—
Adenine	18.834	2.16E-06	5.6651	1.05E-05	—	↓	—	—
Isoleucine	18.58	2.41E-06	5.6178	1.09E-05	—	↓	—	—
Asparagine	17.462	3.95E-06	5.4039	1.49E-05	↓	↓	↑	↑
Methionine	16.765	5.42E-06	5.2659	1.94E-05	—	↓	↑	—
Serine	11.383	8.92E-05	4.0496	0.00030328	—	↓	↑	—
Phenylalanine	10.88	0.00012038	3.9194	0.00038981	—	↓	↑	—
Glutathione	10.464	0.00015511	3.8094	0.00047794	↓	↑	—	—
Valine	10.397	0.00016166	3.7914	0.00047794	—	↓	—	—
Tyrosine	8.094	0.00073852	3.1316	0.0019315	—	↓	—	—
Tryptophan	7.6925	0.00098309	3.0074	0.0024759	—	↓	↑	↑
Threonine	6.3379	0.0027226	2.565	0.0059722	—	—	↑	↑
Glucose-6-phosphate	6.1156	0.0032457	2.4887	0.0068972	—	↓	—	—
Leucine	5.9679	0.0036529	2.4374	0.0075271	—	—	↑	↑
Glycine	5.9079	0.0038338	2.4164	0.0076676	—	↓	—	—
Xanthine	5.7698	0.004288	2.3677	0.0083309	—	↓	—	—
Betaine	5.3657	0.005986	2.2229	0.011001	—	↑	—	—
Acetate	4.6802	0.010768	1.9678	0.018776	—	↓	—	↑
Fumarate	3.9953	0.019921	1.7007	0.03304	↑	↓	—	—
GTP	3.5623	0.029847	1.5251	0.047403	—	—	—	↑
O-Phospho-ethanolamine	3.8036	0.02379	1.6236	0.038517	—	↑	—	—

PC: control pregnant group; PL: pregnant rats fed a 3% leucine-rich diet; PW: pregnant Walker tumor-bearing group; PWL: tumor-bearing pregnant rats fed a 3% leucine-rich diet. Values are presented as *F*-value, *p*-value, and the false discovery rate (FDR). The differences among the groups are indicated by up (increased content) and down (decreased content) arrows.

**Figure 5.**
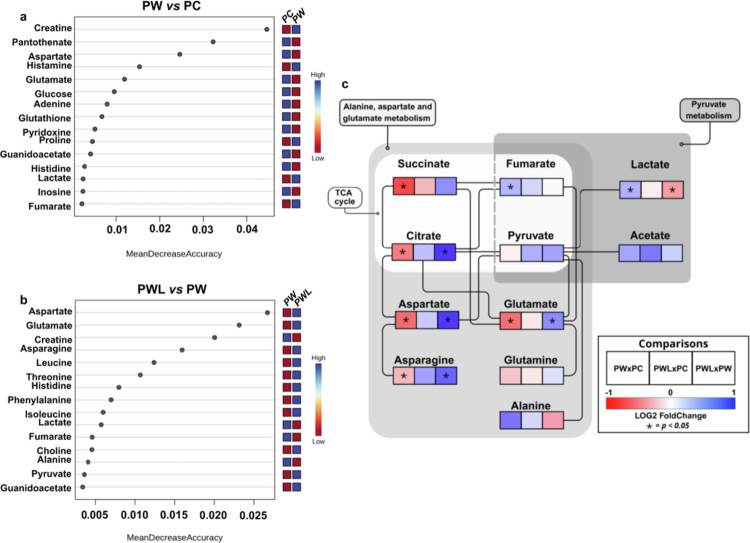
Integrated analysis of metabolomic data in the placental tissue of Walker-256 tumor-bearing rats subjected or not subjected to a leucine-rich diet. (a) Most altered metabolic content in the PW group in comparison to the PC group. (b) Altered metabolite contents in PWL in comparison to PW group. Random forest analysis obtained from MetaboAnalist 6.0 (MetaboAnalyst). (c) Altered metabolic pathways obtained from metabolomic analyses comparing PW *vs* PC, PWL *vs* PC and PWL *vs* PW (comparison in the small squares inside the box, related to the color intensity, varying from the lower (red) to the higher (blue)).

Random forest analysis using MetaboAnalyst identified the main significant metabolites in each comparison (Figure 5a (PW *vs* PC) and 5b (PWL *vs* PW)), and altered metabolic pathways were generated using KEGG database (Supplementary Figure 5). Given the importance of energy metabolism for placental functioning and the possible impairment caused by the tumor influence and the metabolites identified in the random forest analysis, a detailed schematic was constructed focusing on TCA cycle, pyruvate metabolism, and alanine, aspartate, and glutamate metabolism (Figure 5c). In the tumor-bearing group (PW) compared to control group (PC), the key alterations included the upregulation of fumarate and lactate and the downregulation of glucose, aspartate, and glutamate. Conversely, leucine supplementation in tumor-bearing animals (PWL *vs* PW) showed the upregulation of aspartate, glutamate, asparagine, and pyruvate, with the downregulation of lactate, fumarate, and alanine.

### Tumor evolution impacted placenta metabolic pathways, and leucine supplementation could minimize these effects

The metabolomic changes demonstrated variations in cell activity, as evidenced by the metabolites released by the cells, which reflect a real effect on placental tissue. In contrast, the proteomic analysis, which demonstrated a pronounced separation among the experimental groups, suggested that changes in protein profiles had significant metabolic effects that could directly affect the placental metabolomic profile. Integrated proteomic and metabolomic analyses showed that the most significant pathways affected in the placental tissue of the Walker-256 tumor-bearing group (PW) were related to alanine, aspartate, glutamate, and purine metabolism, followed by beta-alanine metabolism and the citrate cycle, compared to the PC group (Table 3), where some particular proteins, such as aldehyde dehydrogenase mitochondrial, Aldehyde dehydrogenase dimeric NADP-preferring, aminopeptidase *N* isocitrate dehydrogenase [NADP] mitochondrial, and citrate synthase mitochondrial were decreased expressed in PW group. Conversely, the leucine-rich diet treatment effect could impact purine metabolism, which is the primary altered pathway in the placental tissue, followed by the metabolism of alanine, aspartate, glutamate, and pyruvate in the PWL group, Compared to PW (Table 3), where the proteins—aldehyde dehydrogenase dimeric NADP-preferring and fumarate hydratase mitochondrial—decreased, but the 6-phosphogluconate dehydrogenase, decarboxylating increased in PWL group, while were closer to PC group. The influence of nutritional supplementation on placental tissue showed interesting points, as these pathways are related to nucleic acid synthesis, ATP production, and the cellular energy balance. These altered metabolites may reflect a metabolic shift toward nucleotide biosynthesis and changes in amino acid metabolism because of leucine supplementation, which plays a role in mTOR activation and protein synthesis.

**Table 3.** Integrated analysis of proteomic and metabolomic data showing the impacted pathway in the placenta tissue of *Wistar* pregnant *Walker*-256 tumor-bearing rats, subjected or not to 3% leucine-rich diet, and compared to control groups.

		Alanine, aspartate, and glutamate metabolism			
		PW *vs* PC	PL *vs* PC	PWL *vs* PW	PWL *vs* PC
Compounds	Alanine	**—**	**—**	**—**	**—**
	Asparagine	↓	↓	↑	↑
	Aspartate	↓	**—**	↑	**—**
	Fumarate	↑	↓	**—**	**—**
	Glutamate	↓	**—**	↑	**—**
	Succinate	↓	↑	**—**	**—**
Genes	Ass1 (P09034)	**—**	↓	**—**	**—**
		Histidine metabolism			
		PW *vs* PC	PL *vs* PC	PWL *vs* PW	PWL *vs* PC
Compounds	Aspartate	↓	**—**	↑	**—**
	Creatine	↑	**—**	**—**	**—**
	Glutamate	↓	**—**	↑	**—**
	Histamine	↑	**—**	**—**	↑
				**—**	
Genes	Aldh2 (P11884)	↓	**—**	**—**	↓
	Aldh3a1 (P11883)	↓	**—**	↓	↓
		Glutathione metabolism			
		PW *vs* PC	PL *vs* PC	PWL *vs* PW	PWL *vs* PC
	Glutamate	↓	**—**	↑	**—**
	Glutathione	↓	↑	**—**	**—**
	Glycine	**—**	↓	**—**	**—**
Genes	Anpep (P15684)	↓	**—**	**—**	**—**
	Idh2 (P56574)	↓	**—**	**—**	**—**
	Pgd (P85968)	**—**	**—**	↑	**—**
	Gclm (P48508)	**—**	**—**	**—**	↓
		Citrate cycle (TCA cycle)			
		PW *vs* PC	PL *vs* PC	PWL *vs* PW	PWL *vs* PC
Compounds	Fumarate	↑	↓	↓	**—**
	Succinate	↓	↑	↑	**—**
Genes	Idh2 (P56574)	↓	**—**	**—**	**—**
	Cs (Q8VHF5)	↓	**—**	**—**	**—**
	Fh (P14408)	**—**	**—**	↓	**—**
	Dld (Q6P6R2)	**—**	**—**	**—**	↓

PC: control pregnant group; PL: pregnant rats fed a 3% leucine-rich diet; PW: pregnant Walker tumor-bearing group; PWL: tumor-bearing pregnant rats fed a 3% leucine-rich diet. Integrated analysis was performed from metabolomic and proteomic profiles using the MetaboloAnalyst platform (MetaboAnalyst 6.0. https://www.metaboanalyst.ca/MetaboAnalyst/). For details, see Material & methods. The differences among the groups are indicated by up (increased content) and down (decreased content) arrows.

## Discussion

The pregnancy course is a complex and delicate process that can be affected by pathological conditions such as cancer. Therefore, tumor evolution can negatively impact placental development and function, directly affecting the health of fetuses, as well as the health of the mother. In this way, the present study provides new insights into the complex association between tumor progression, particularly in the Walker-256 tumor model, and gestational development, as well as the impact of nutritional intervention with a leucine-rich diet. Few studies in the literature address this topic comprehensively, as proteomic and metabolomic levels are associated with affected pathways, providing a detailed view of placental tissue activity in a cancer-cachexia state. In this way, as an innovative approach, we demonstrated some potential for intervention through leucine supplementation, which alleviated some of the negative impacts resulting from tumor evolution.

The presence of a tumor led to substantial reductions in maternal weight, as well as the growth and development of the placenta and, consequently, the fetus. These physiological impairments are consequences of alterations in proteomic and metabolomic profiles, revealing dysregulation of protein synthesis and expression and metabolites related to energy metabolism, cellular structure, and communication in the placental tissue. These results showed negative impacts on essential pathways, such as protein folding, amino acid synthesis, and the citrate cycle. These findings are consistent with prior studies on cancer-associated cachexia and metabolic wasting during pregnancy,^8^^,^^47^ which showed that tumor evolution causes intense maternal body weight loss, imposing negative effects on the pregnancy course through maternal health impairment, as shown here, in addition to damaging maternal host tissues, including placental tissue.^1^^,^^8^^,^^14^^,^^48-50^ Interestingly, while fetal weight was reduced by tumor evolution, there was a partial protective effect of leucine supplementation on placental function and, consequently, minimizing the negative effect on fetal weight. Most of the placental proteins and metabolites change, leading to alterations in pathways, which are reflected in impaired cell activity and metabolism under the effects of tumors. These results are in consonance with the affected biological processes (GO), as most pathways in the tumor-bearing group were related to cellular structure and organization, energy metabolism, and protein processing, with a considerable reduction in protein synthesis. To our knowledge, no studies have examined these specific placental modulations in the context of cancer, highlighting the novelty of our findings. In addition to the main altered proteins, our comparison between the two tumor-bearing groups and the control group revealed the most significant correlations between proteomic and metabolomic data in the placental tissue. The observed alterations were especially prominent in key metabolic pathways, such as alanine, aspartate, and glutamate metabolism, histidine metabolism, glutathione metabolism, and the tricarboxylic acid (TCA) cycle—all of which highlighted the positive impact of nutritional supplementation. Integrated proteomic and metabolomic analysis demonstrated that the presence of a tumor likely compromised placental integrity at multiple levels: structural (cytoskeletal proteins), functional (mitochondrial and transporter proteins), and metabolic (reduction of essential substrates and energy cofactors), imposing damage to fetal development.

In previous studies, tumor evolution led to negative effects on pregnant rats, with a deep impact on fetal development, showing intrauterine growth restriction (IUGR), which was associated with alterations in many metabolic pathways in maternal serum, placenta and fetal tissues.^8^ The authors suggested that not only did the tumor cause inefficiency of placental glucose metabolism, but also led to a negative impact on amino acid and glycerophospholipid metabolism pathways, which contributed to the reduction in fetal weight,^8^ although the authors emphasized that these changes could be caused by tumor growth during early pregnancy affecting the placenta and also by a direct effect of the tumor growth itself. Maternal protein deprivation studies in rats, as well as during tumor evolution, which can lead to nutritional failure, have shown downregulation of placental amino acid transporters^8^^,^^21^^,^^50-53^, where the reduction in total amino acid concentrations can be related to alterations in amino acid transporter systems in human and rodent placentas, which may be similar to that observed in the PW group. Supporting this, other previous studies demonstrated that factors and cytokines released by tumor or host cells jeopardized the pregnancy course, impairing the placenta and fetal development.^14^^,^^48^^,^^49^ These findings suggest that altered placental cellular function may be directly linked to tumor-associated damage. The placental amino acid metabolism proceeds through major pathways that utilize carbon and nitrogen accretion for fetal growth, interconversion into other substrates, or use them as oxidative fuels for the placenta or fetus.^8^^,^^48^^,^^49^^,^^51^^,^^52^ In a physiological state, the placenta is a highly metabolically active organ, where the consumption of O_2_ and glucose is high, and maternal metabolic adaptations ensure sufficient supply to the growing fetus. These adaptations include rendering the mother's tissues transiently insulin-resistant and enhancing maternal glucose production by 30%, from early to late pregnancy.^8^^,^^21^^,^^48^^,^^49^^,^^51^^,^^52^ Healthy placental development is crucial to the pregnancy course, and placental insufficiency often leads to miscarriage, IUGR, and preeclampsia^6^^,^^48^—as evidenced in the present study by the association between cancer and pregnancy.

Tumor-related disturbances in the metabolic profile led to alterations in energy balance, marked by reductions in adenosine, aspartate, glutamate, succinate, and glucose levels, like the findings of Salgado and colleagues.^8^ These alterations are consistent with mitochondrial dysfunction, oxidative stress, and inhibition of the Krebs cycle—features typically observed in cachectic tissues.^54^ Additionally, the accumulation of the placental contents of lactate, creatine, and histamine suggested a metabolic shift towards anaerobic and inflammatory pathways, further reinforcing bioenergetic impairment, as observed under the negative effect of tumor growth. Since alanine, aspartate, and glutamate metabolism play a central role in nitrogen homeostasis, oxidative stress regulation, and mitochondrial function, their involvement in placental physiology is expected.^55^ In the tumor-bearing group (PW), the observed depletion of these amino acids likely indicates mitochondrial dysfunction and increased nitrogen demand—two factors that may impair placental bioenergetics and biosynthetic capacity.^8^^,^^56^^,^^57^ As histidine plays an important role in inflammation and antioxidant defense, modulating the vascular permeability and immune responses in the placenta,^58-60^ the enhanced histidine content in PW group may suggest a pro-inflammatory state and impaired antioxidant defense in placental tissue,^56^^,^^57^ as observed in other pathologies such as preeclampsia and gestational diabetes.^61-64^

Remarkably, leucine supplementation (PWL) partially restored the levels of most placental metabolites, particularly adenosine, pyridoxine, glutamate, and aspartate, which likely indicated an improvement in oxidative metabolism and a reduction in cellular stress. Furthermore, essential metabolic pathways for placental bioenergetics and redox regulation were significantly impacted by tumor development. The reduced flux through the tricarboxylic acid (TCA) cycle, evidenced by decreased succinate and glucose and increased lactate, reflects a shift toward anaerobic metabolism, a hallmark of mitochondrial stress, characteristic of cachectic and hypoxic tissues.^8^^,^^48^^,^^49^ The increase in placental NAD⁺ and ATP content driven by the effect of leucine supplementation may stimulate the cellular bioenergetics, possibly via activation of the mTOR and anti-catabolic process, as verified in our previous studies.^13^^,^^15^^,^^17^^,^^19-21^ Additionally, there is a need for more studies to investigate these specific placental modulations in the context of cancer under nutritional supplementation.

Leucine supplementation appeared to be effective in mitigating some of the negative effects caused by tumor progression in the PWL group, particularly regarding proteomic profiles, such as some key proteins that were notably altered by the tumor presence, such as zinc-alpha-2-glycoprotein (ZAG), tubulin beta-4B chain (Tubb4B), and cystatin C. The PWL group exhibited fewer alterations in these proteins as compared to the PW group. The tumor-induced alteration in ZAG in the placental tissue may be affecting placental lipid metabolism and energy supply, although this protein is traditionally studied in cancer as a biomarker.^65-67^ There are still no studies linking ZAG to placental function under tumor influence, nor under leucine supplementation, which may affect the placental metabolic regulation and immune modulation during pregnancy.^68-71^ Leucine supplementation has brought the synthesis of MAGI2 closer to the control group, suggesting that it promoted more stable placental development without the overstimulation caused by tumor progression. Conversely, as a damaging effect of tumor evolution, the decreased tubulin beta-4B chain (Tubb4B) in the rat placenta could be related to an impaired cytoskeletal function.^72^ Therefore, the diminished expression could interfere with microtubule function, which jeopardizes the correct development and function of placental cells, negatively impacts cellular proliferation, and harms fetal development, as seen here and in our previous work.^14^^,^^15^^,^^48-50^^,^^53^ Although knowledge about MAGI2 and TUBB4B in placental tissue is limited, we suggest that alterations in these proteins could affect placental development during tumor progression through impairment of cell signaling and structural integrity. Additionally, when comparing both tumor-bearing groups (PWL *vs* PW), the three most altered proteins—cystatin-C, translocon-associated protein subunit alpha, and destrin—showed reduced placenta content in PWL. Some studies have reported that the increased concentration of cystatin C protein in the placenta is related to severe pre-eclampsia, which is supported by its presence in amniotic fluid samples.^73-76^ In the PWL group, the reduced placental content of cystatin C protein may indicate a protective effect against tumor-induced dysfunction, improving placental weight and leading to better fetal development. Furthermore, as the translocon-associated protein subunit alpha is potentially involved in glucose metabolism and energy supply^77^^,^^78^ and, as a destrin, plays a significant role in embryonic development^79^^,^^80^—also participating in cell structure organization and migration during cancer progression^81-83^—it is plausible to suggest that these protein alterations are related to the beneficial effects of a leucine-rich diet.

Moreover, comparing the placental tissue during tumor evolution modulated by leucine nutritional supplementation, the correlation of proteomic and metabolomic data revealed changes in the placental biological pathways related to alanine, aspartate, glutamate, histidine, and glutathione metabolism, as well as citrate cycle synthesis, which integrate the changed metabolites with some proteins/genes, as shown in Table 3. As mentioned above, these impacted pathways are essential for healthy placental function and, under the influence of tumors, could be deeply affected. However, the nutritional supplementation showed benefits, especially in minimizing the impact on alanine, aspartate, and glutamate metabolism, as well as glutathione metabolism, bringing these pathways closer to those in the control group. This likely improved placental metabolism in the PWL group, which was associated with increased energy availability through the citrate cycle, also like the control group. Interestingly, the leucine supplementation resulted in fewer proteins and metabolites being differentially expressed, and most of them were closer to those in the control group, resulting in a shift in the pathways affected in PW towards amino acid biosynthesis and pyruvate metabolism, which suggests a more regulated or adaptive metabolic response in the placenta. To the best of our knowledge, this study has shown, for the first time, some important findings about the influence of nutritional supplementation on tumor-induced damage in placental tissue.

Further studies are currently underway in our laboratory to investigate whether, and through which mechanisms, leucine supplementation may exert modulatory molecular effects on pregnancy-associated cancer. Despite having the availability of various breast cancer models, as breast cancer represents the most common malignancy diagnosed in pregnant patients,^84^ to our knowledge, there is no breast cancer model to be used to mimic cancer during pregnancy, which represents a significant limitation. As we have published previous results and given the significant lack of studies on cancer during pregnancy, especially in placental tissue, it is important to analyze the effects of cancer development in a general context. Given the rare incidence of cancer during pregnancy, the cachexia development could occur at a very low probability, and considering that the most common cancers, as breast and cervical, diagnosed during pregnancy, there is a remarkable potential for underestimation. In this way, as the Walker-256 tumor is a robust pre-clinical model of cachexia, all the comparisons of the alterations found in the association of cancer during pregnancy should be worth translating to the clinical settings. Nevertheless, our findings demonstrate that a leucine-rich diet can attenuate some of the harmful effects of tumor progression, providing valuable insights. These findings could establish a basis for further research into leucine supplementation as a potential adjuvant strategy for the association between cancer and pregnancy.

## Conclusion

In summary, this pre-clinical study shows that the Walker-256 tumor negatively impacts the pregnancy course, affecting the placental activity and leading to lower fetal and placental weights, as well as higher rates of fetal resorption. On the other hand, a leucine-rich diet has a partial, but positive effect on the placenta of tumor-bearing rats, partially improving placental activity and minimizing fetal weight loss and resorption. These findings highlight the potential use of leucine supplementation as an adjunct therapy in the context of maternal metabolic stress during tumor growth.

## Supplementary Material

Supplemental Material.docx Supplemental Material.docx

Supplemental Material ARRIVE_guidelines_Author_Checklist_Santos_et_al.pdf

## Data Availability

Data will be made available on reasonable request.
