# Supplementary material for: Impact of leucine-rich diet on placenta's proteomic and metabolomic profiles of pregnant tumor-bearing rats
Source: Cancer Biol Ther. 2026 May 14;27(1):2670792. doi: 10.1080/15384047.2026.2670792 (PMC13182973; doi:10.1080/15384047.2026.2670792)
Supplement: Supplemental Material.docx [file KCBT_A_2670792_SM1723.docx]

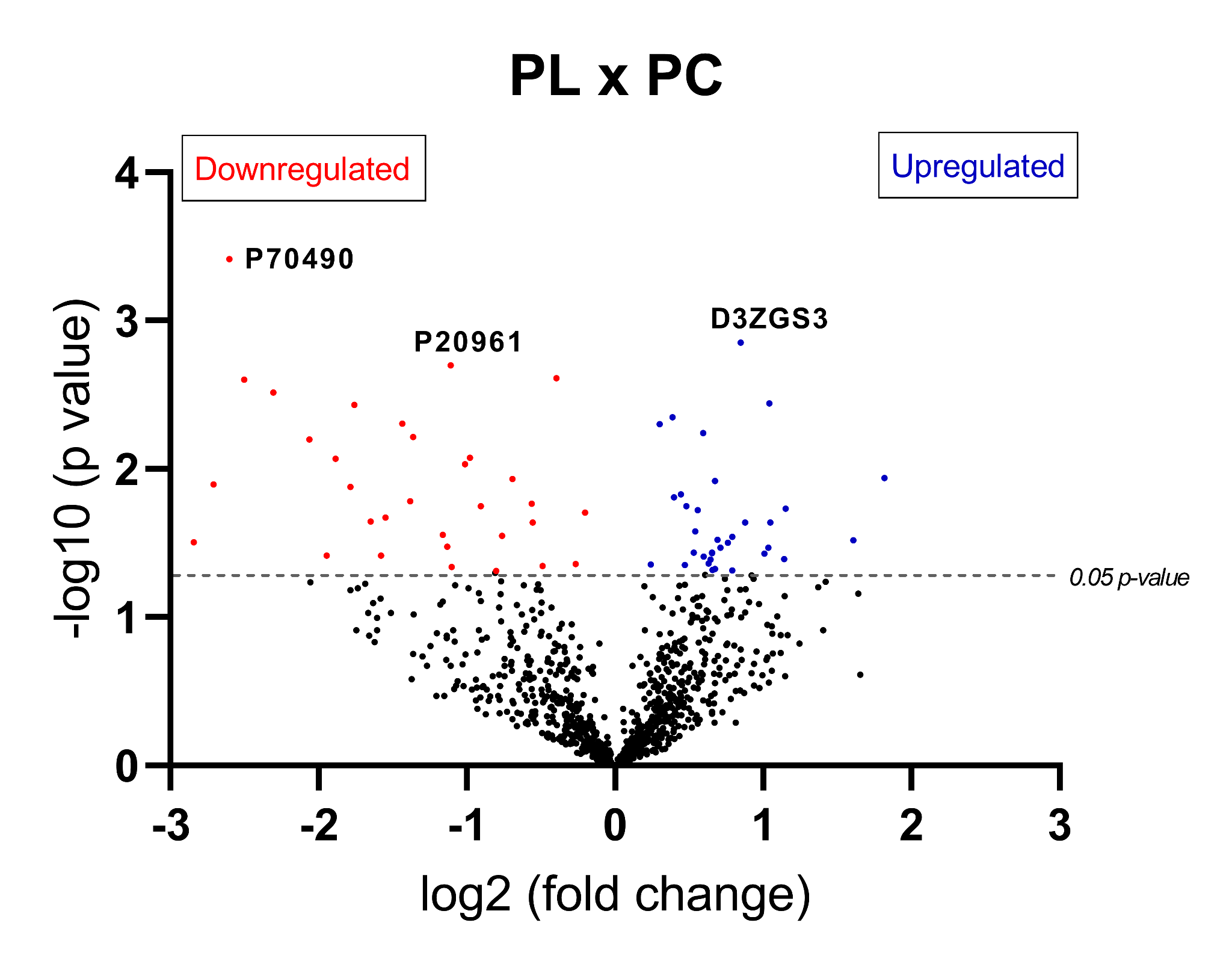


**Figure Supplementary 1: Volcano Plot Comparison between PL vs PC groups.** Volcano plots illustrating differentially expressed proteins between the comparison PL vs PC group. Fold-change values were converted to a base 2 logarithm, and p-values to a base -10 logarithm. The horizontal line indicates the significance threshold (p-value in -log10 ≥ 2), distinguishing significantly altered proteins from non-significant ones. Proteins with decreased expression are displayed on the left, while those with increased expression are shown on the right. Legend: PC (Control), PL (Leucine).


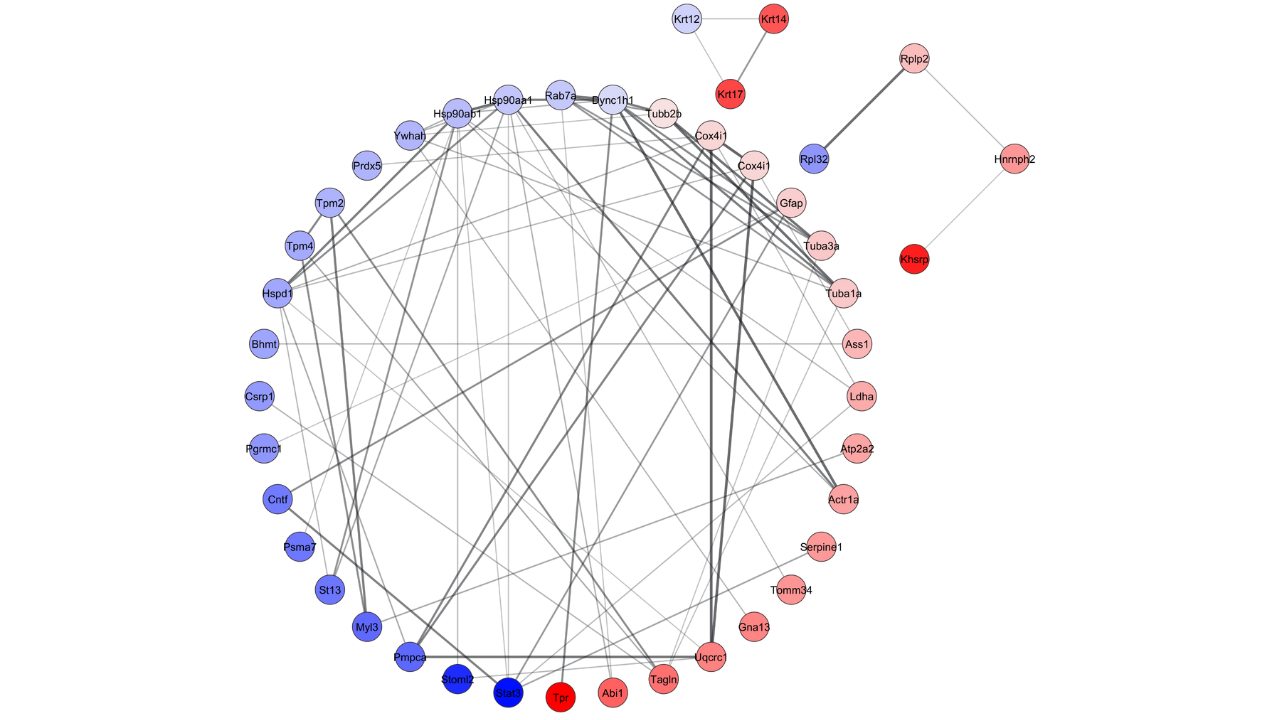


**a) PW *vs* PC**

**b) PL *vs* PC**


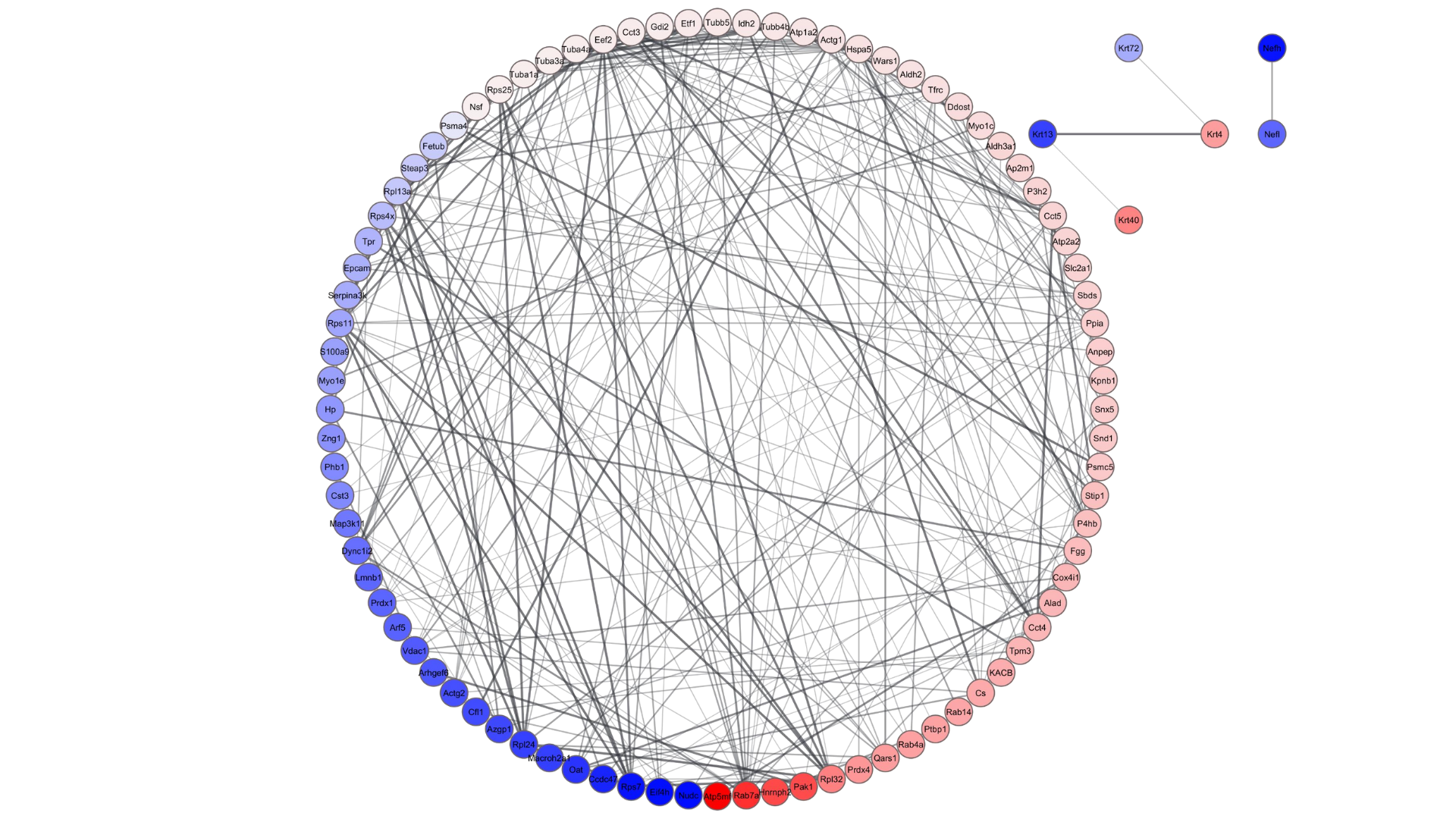


**c) PWL *vs* PW**


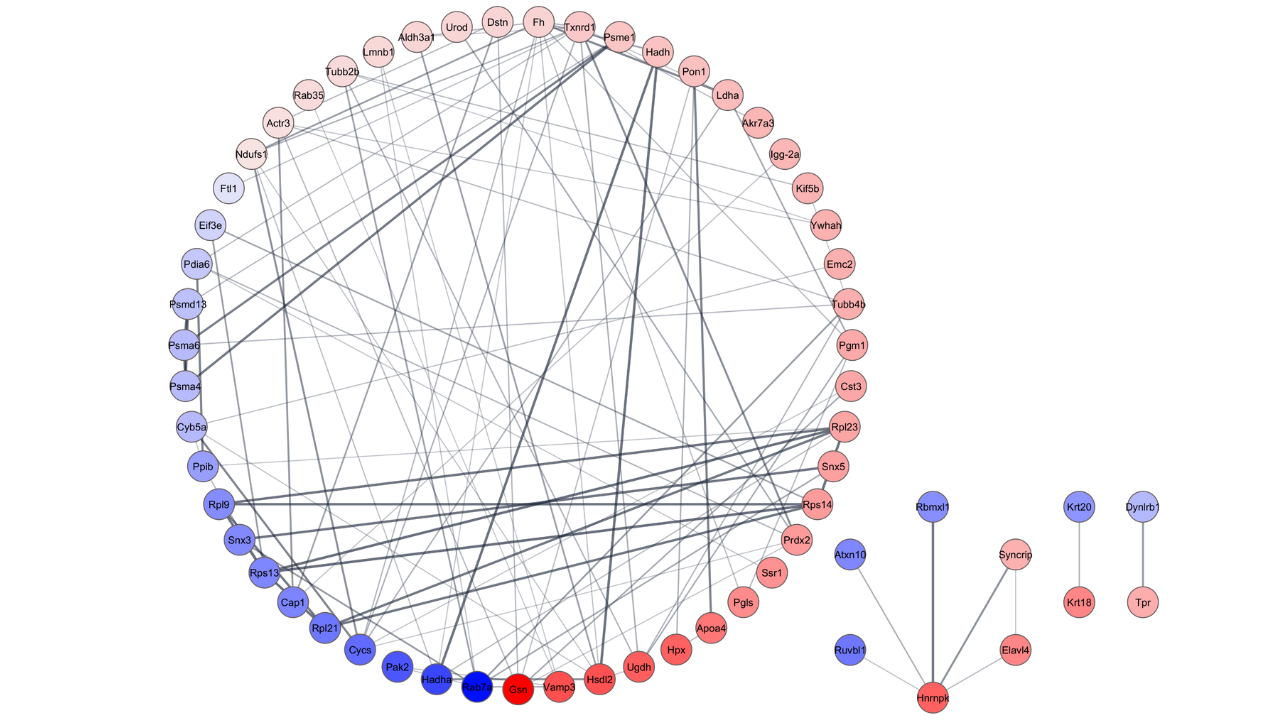


**Supplementary Figure 2: Protein-Protein Interaction (PPI) networks among the groups.** PPI networks of significantly altered placental proteins using STRING and Cytoscape. Unconnected proteins were removed. The colours and intensity represent the direction and magnitude of regulation: red nodes are downregulated and blue nodes are upregulated, with darker tones representing higher fold change values. **a**: Comparison between PW (Walker tumour-bearing group of pregnant rats) and PC (control group of pregnant rats); **b**: Comparison between PL (pregnant rats fed a 3% leucine-rich diet) and PC; **c**: Comparison between PWL (tumour-bearing pregnant rats fed a 3% leucine-rich diet) and PW. Legend: PC (Control), PL (Leucine), PW (Walker-256), and PWL (Walker-256 + Leucine).

**a) PL *vs* PC**


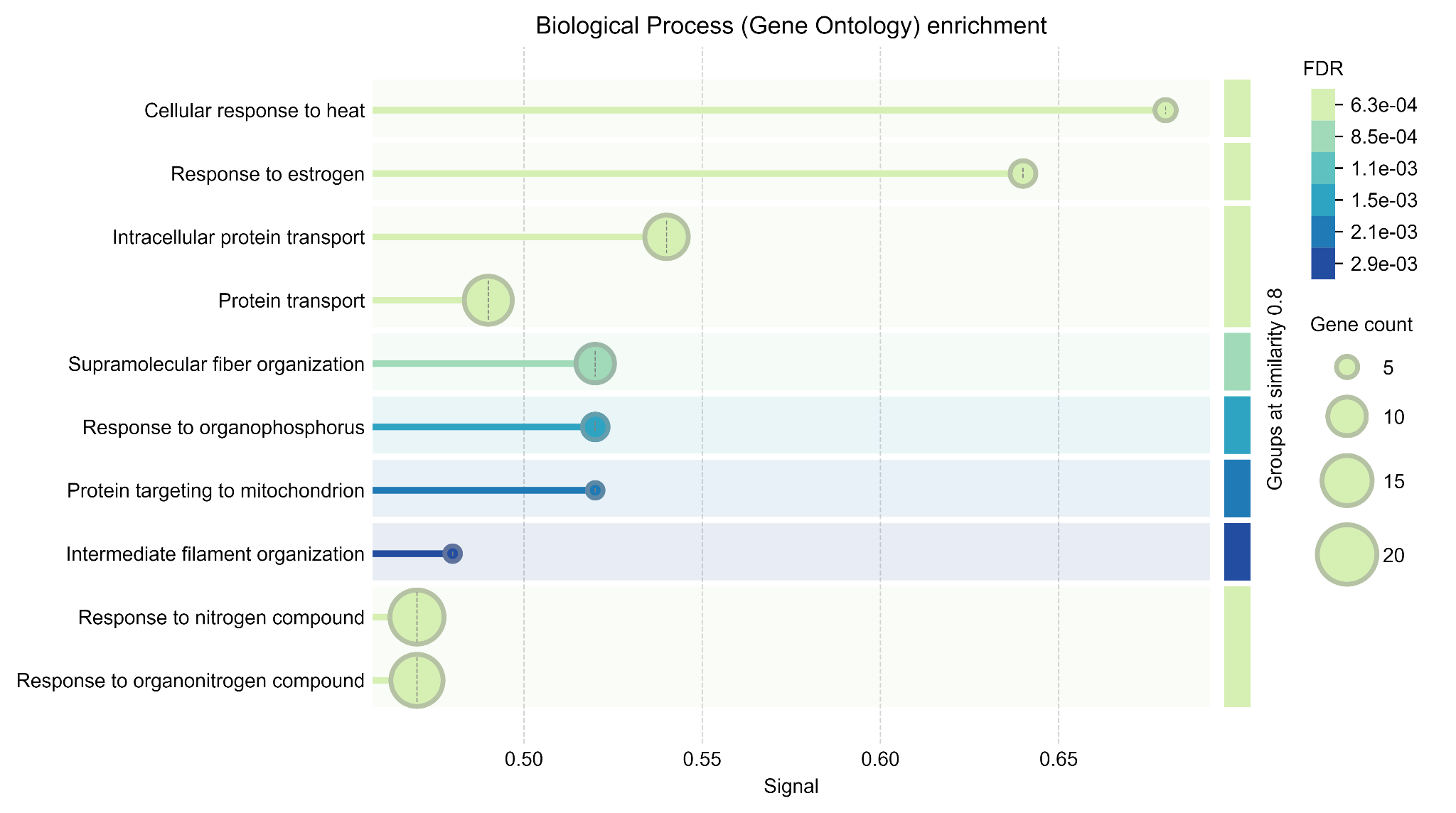


**b)**


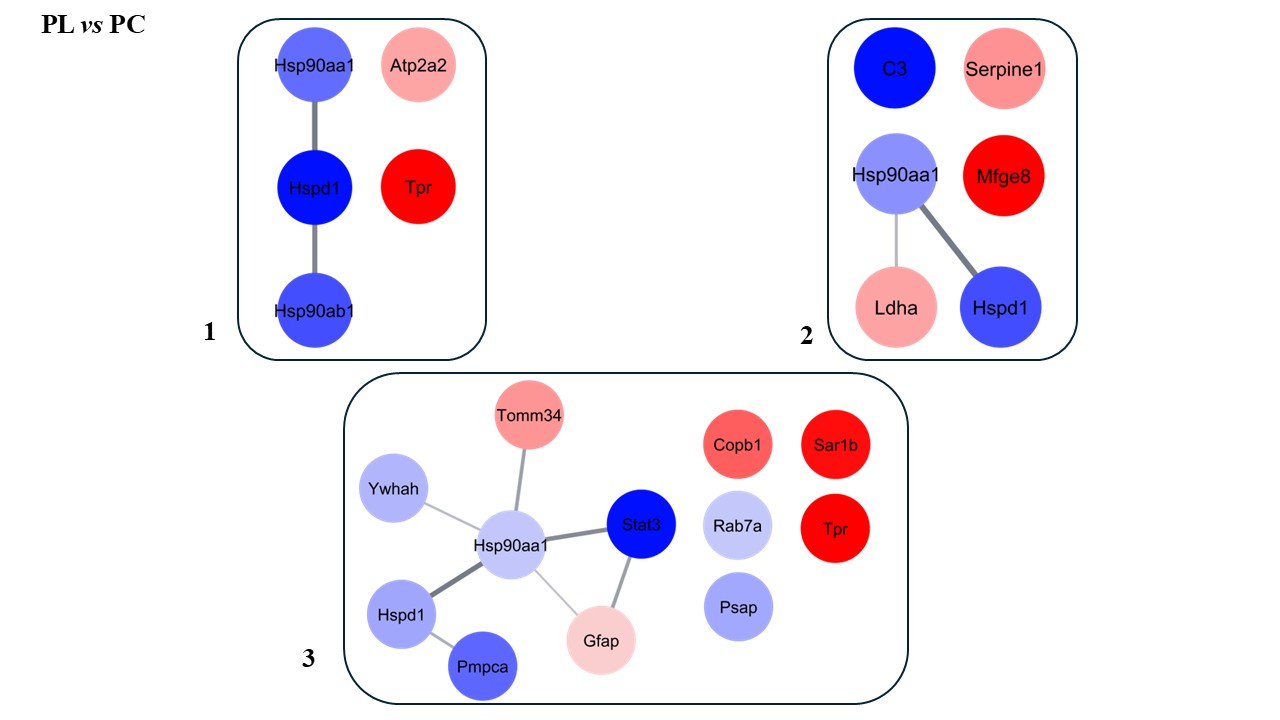


**Supplementary Figure 3:** **Protein networks and biological pathways were impacted in placental tissue from tumour-bearing rats, subject to or not to a leucine-rich diet. (a)** Pathways with higher impact (Signal) (top) show the altered biological processes from The Gene Ontology Database pathways (GO) in the healthy group under a leucine-enriched diet (PL) compared to the control (PC). Data derived from PPI networks generated in String. Pathways are ranked by pathway signal value; a weighted harmonic mean balancing significance (-log(FDR)) and enrichment (observed/expected ratio). The scale on the right displays False Discovery Rate (FDR) values, while dot sizes represent the raw number of altered proteins. **(b)** The three GO pathways with the highest signal values (in the top) in PL compared to PC. Node colours indicate the type of regulation (red: decreased synthesis; blue: increased synthesis), with colour intensity dependent on the FoldChange value. (1) Cellular response to heat; (2) Response to estrogen; (3) Intracellular protein transport.

**.**
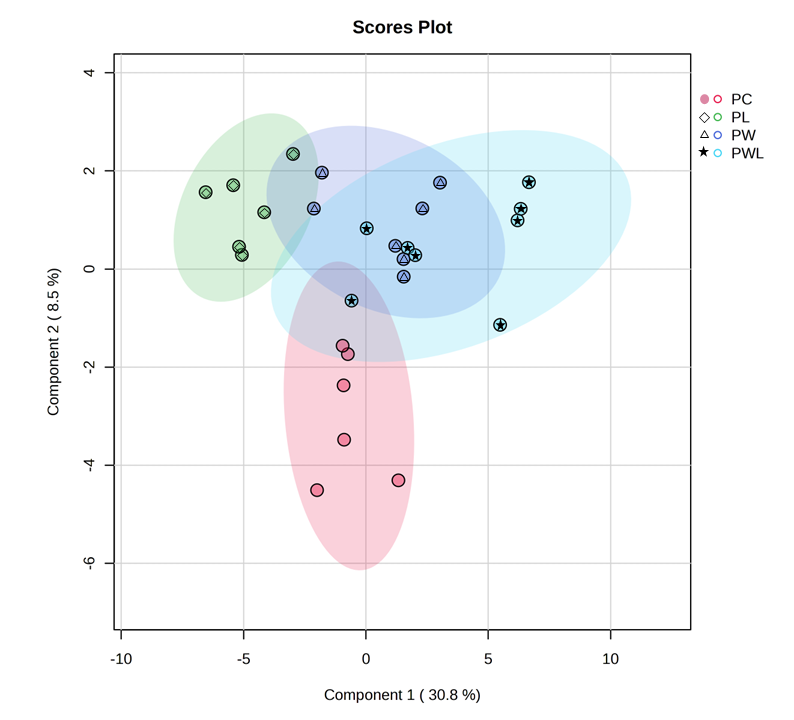


**Supplementary Figure 4: Proteomic profile in placenta tissue from different experimental groups, implanted with Walker-256 tumour cells and subject to or not to leucine-rich diet.** The principal components reveal a reasonable separation between the groups, with a slight overlap between the data of the tumour groups with and without leucine. PLSDA - Score Plot analysis of the proteomics data from the placentas. The experimental groups are represented by colours: Control (PC, red), Walker-256 (PW, blue), Leucine (PL, green), and Walker-256 + Leucine (PWL, purple).


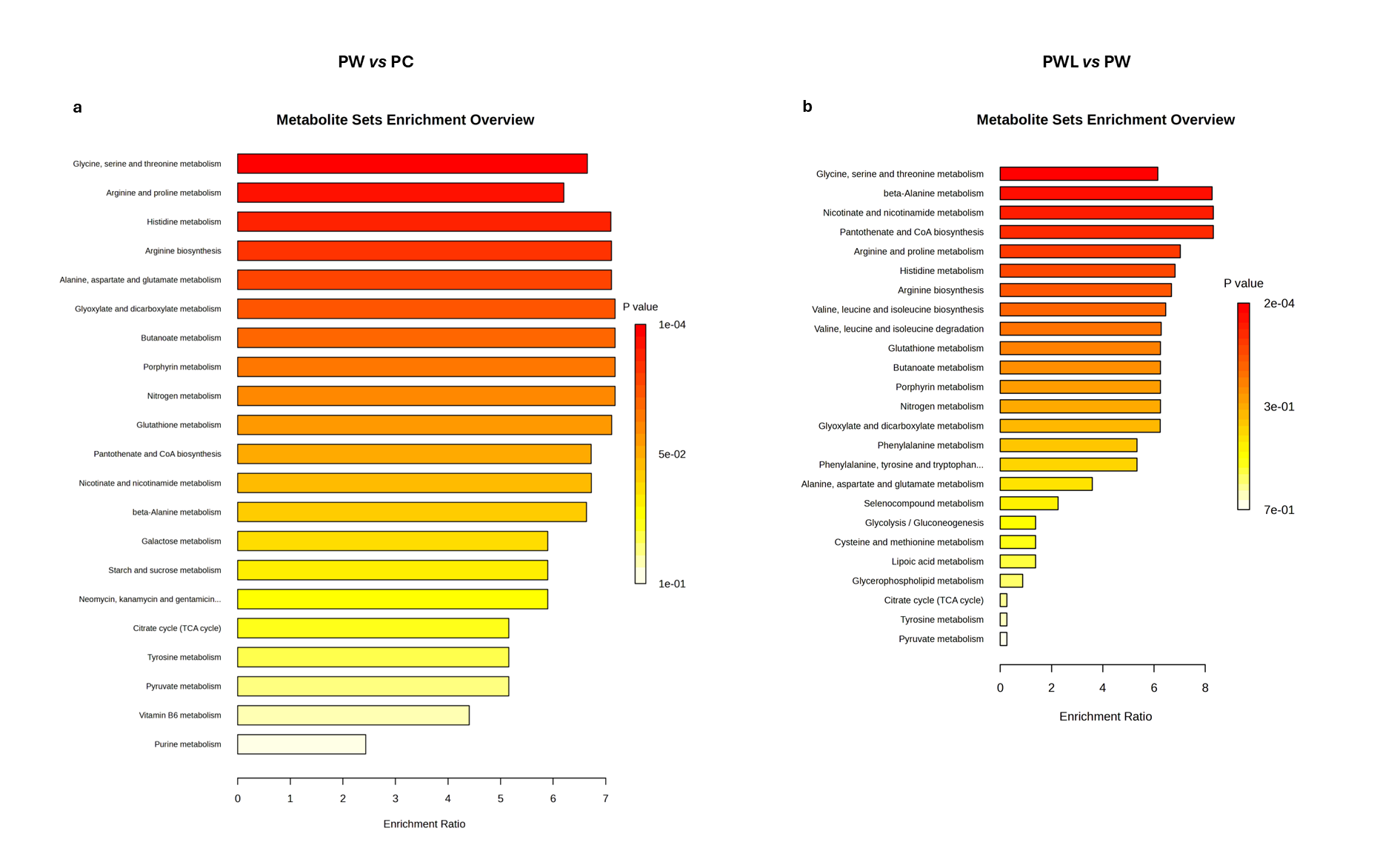


**Supplementary Figure 5: KEGG pathways derived from the main significantly altered metabolites.** Data generated from the concentrations of significant metabolites identified in the Random Forest analysis, via MetaboAnalyst. Pathways with the greatest impact (most significant p-value; p < 0.05) are at the top. The x-axis presents the Enrichment Rate values. **(a)** Walker-256 tumour-bearing group (PW) compared to the control group (PC); **(b)** supplemented Walker-256 tumour-bearing group (PWL) compared to the non-supplemented Walker-256 tumour-bearing group (PW).

| Supplementar Table 1. Proteins Concentration Datasheet | | | |  |
| --- | --- | --- | --- | --- |
| **Proteins** | **PC** | **PL** | **PW** | **PWL** |
|  | **Mean ± SD (x10^6)** | **Mean ± SD (x10^6)** | **Mean ± SD (x10^6)** | **Mean ± SD (x10^6)** |
| B0BN93 | 2.23 ± 1.02 | 4.01 ± 2.20 | 2.30 ± 1.26 | 4.56 ± 4.92^#^ |
| B0BNG0 | 4.54 ± 4.13 | 1.49 ± 5.98 | 6.56 ± 1.36 | 3.04 ± 3.87^#^ |
| B1H267 | 4.00 ± 1.68 | 2.67 ± 2.54 | 2.40 ± 9.59* | 0.95 ± 0.74^#^ |
| D3ZAF6 | 4.61 ± 2.55 | 4.64 ± 2.04 | 0.26 ± 6.60* | 6.06 ± 1.56 |
| D3ZGS3 | 4.57 ± 1.98 | 8.24 ± 1.01* | 2.45 ± 2.76 | 5.45 ± 4.55 |
| D4AE41 | 4.20 ± 3.79 | 2.66 ± 3.27 | 1.50 ± 9.64 | 5.48 ± 3.00^#^ |
| F1MA98 | 4.95 ± 4.21 | 0.69 ± 4.57* | 8.75 ± 0.85* | 3.94 ± 1.14^#^ |
| O09032 | 4.78 ± 2.52 | 3.26 ± 3.24 | 4.29 ± 4.84 | 1.27 ± 1.59^#^ |
| O09171 | 1.99 ± 5.39 | 3.18 ± 9.61* | 1.34 ± 1.44 | 3.58 ± 3.30 |
| O09178 | 3.58 ± 0.64 | 3.35 ± 1.57 | 2.96 ± 0.19* | 2.73 ± 2.28 |
| O35814 | 3.56 ± 2.50 | 2.85 ± 1.47 | 1.85 ± 0.10* | 1.92 ± 1.09 |
| O55159 | 1.53 ± 6.81 | 2.08 ± 7.24 | 2.77 ± 4.66* | 3.61 ± 2.93 |
| O70199 | 4.32 ± 2.97 | 5.03 ± 3.67 | 6.54 ± 1.17 | 1.29 ± 1.29^#^ |
| O70535 | 1.99 ± 2.58 | 2.45 ± 8.69 | 0.79 ± 8.76* | 1.91 ± 0.93 |
| O88382 | 1.89 ± 2.88 | 3.02 ± 3.40 | 8.96 ± 2.70* | 3.47 ± 7.28^#^ |
| O89049 | 4.07 ± 3.27 | 5.08 ± 4.14 | 4.94 ± 1.35 | 2.97 ± 3.03^#^ |
| P00173 | 1.74 ± 1.14 | 2.78 ± 1.00 | 2.04 ± 1.07 | 4.38 ± 3.46^#^ |
| P00406 | 6.82 ± 2.88 | 3.91 ± 1.69* | 5.08 ± 5.54 | 3.78 ± 2.76 |
| P01026 | 4.95 ± 3.84 | 9.09 ± 4.30* | 2.93 ± 3.31 | 6.73 ± 5.72 |
| P01048 | 5.96 ± 3.28 | 7.66 ± 3.16 | 1.50 ± 1.36* | 8.00 ± 5.53 |
| P01835 | 5.65 ± 3.50 | 2.65 ± 3.09 | 2.34 ± 6.61* | 7.38 ± 4.45^#^ |
| P02401 | 3.42 ± 3.59 | 2.12 ± 1.19* | 3.54 ± 8.53 | 2.37 ± 2.13 |
| P02651 | 6.42 ± 4.20 | 4.21 ± 3.87 | 9.11 ± 1.07 | 2.32 ± 1.79^#^ |
| P02680 | 3.57 ± 1.30 | 4.56 ± 2.48 | 1.79 ± 2.16* | 1.41 ± 1.02 |
| P02793 | 6.33 ± 1.93 | 7.61 ± 2.17 | 6.97 ± 1.04 | 8.99 ± 6.39^#^ |
| P04182 | 1.87 ± 1.19 | 2.54 ± 3.61 | 9.19 ± 4.25* | 1.47 ± 1.35 |
| P04642 | 7.27 ± 1.24 | 3.88 ± 3.23* | 7.23 ± 0.94 | 3.82 ± 7.62^#^ |
| P04785 | 2.77 ± 1.01 | 2.39 ± 1.15 | 1.44 ± 1.64* | 1.75 ± 1.52 |
| P04937 | 4.34 ± 1.72 | 3.52 ± 1.43 | 3.72 ± 2.43 | 1.95 ± 1.80^#^ |
| P05197 | 3.88 ± 7.06 | 3.50 ± 1.76 | 3.45 ± 2.99* | 3.02 ± 2.89 |
| P05544 | 1.92 ± 2.65 | 2.46 ± 2.84 | 4.74 ± 5.68* | 8.27 ± 5.86 |
| P05545 | 2.28 ± 2.89 | 2.48 ± 2.60 | 4.27 ± 5.90* | 6.56 ± 4.82 |
| P05714 | 6.27 ± 2.08 | 5.81 ± 3.25 | 2.25 ± 2.70* | 2.34 ± 2.43 |
| P06214 | 3.48 ± 8.96 | 2.70 ± 1.32 | 1.61 ± 1.89* | 1.55 ± 1.09 |
| P06686 | 4.32 ± 1.45 | 3.59 ± 1.91 | 3.53 ± 5.26* | 1.88 ± 1.11 |
| P06761 | 4.93 ± 1.24 | 4.18 ± 4.77 | 3.85 ± 3.66* | 4.70 ± 4.63 |
| P06866 | 2.73 ± 2.32 | 3.86 ± 2.64 | 6.14 ± 0.54* | 4.61 ± 2.21 |
| P07871 | 1.53 ± 1.20 | 1.49 ± 9.52 | 9.32 ± 6.92* | 2.44 ± 9.04 |
| P09005 | 3.60 ± 8.92 | 4.91 ± 0.79* | 1.54 ± 1.86 | 5.28 ± 4.39 |
| P09034 | 5.46 ± 1.98 | 3.22 ± 1.07* | 3.81 ± 1.73 | 3.97 ± 3.92 |
| P09495 | 1.28 ± 7.26 | 1.98 ± 2.34* | 1.57 ± 8.17 | 1.81 ± 1.62 |
| P09527 | 3.84 ± 3.28 | 5.02 ± 8.91* | 0.37 ± 0.23* | 5.87 ± 4.02^#^ |
| P09626 | 4.57 ± 1.71 | 4.49 ± 3.00 | 2.73 ± 7.04* | 1.20 ± 1.24 |
| P10111 | 7.62 ± 8.03 | 6.95 ± 1.81 | 4.74 ± 6.56* | 4.50 ± 3.38 |
| P10688 | 3.29 ± 0.48 | 3.36 ± 1.19 | 2.25 ± 0.31* | 1.43 ± 1.94 |
| Supplementar Table 1. Proteins Concentration Datasheet (Continuation) | | | |  |
| **Proteins** | **PC** | **PL** | **PW** | **PWL** |
|  | **Mean ± SD (x10^6)** | **Mean ± SD (x10^6)** | **Mean ± SD (x10^6)** | **Mean ± SD (x10^6)** |
| P10888 | 7.59 ± 8.66 | 5.77 ± 9.07* | 3.53 ± 4.08* | 2.01 ± 9.83 |
| P10960 | 1.39 ± 7.75 | 2.16 ± 3.35* | 2.10 ± 0.02 | 1.18 ± 1.53 |
| P11167 | 5.51 ± 1.80 | 4.67 ± 3.01 | 3.59 ± 4.71* | 2.36 ± 2.33 |
| P11507 | 6.26 ± 1.71 | 3.18 ± 1.95* | 4.18 ± 8.97* | 1.97 ± 1.76 |
| P11883 | 4.67 ± 1.37 | 3.26 ± 1.08 | 3.18 ± 5.16* | 2.24 ± 2.01^#^ |
| P11884 | 3.37 ± 7.96 | 3.87 ± 8.50 | 2.62 ± 4.15* | 1.00 ± 0.22 |
| P12788 | 5.89 ± 2.56 | 2.74 ± 2.69* | 1.46 ± 3.21 | 2.81 ± 1.95 |
| P13471 | 3.79 ± 3.18 | 5.10 ± 3.11 | 6.04 ± 1.12 | 2.26 ± 1.84^#^ |
| P13676 | 5.65 ± 2.85 | 1.14 ± 9.47* | 3.97 ± 4.14* | 3.30 ± 8.34 |
| P14408 | 3.51 ± 2.47 | 3.79 ± 3.01 | 2.97 ± 7.20 | 1.99 ± 1.56^#^ |
| P14841 | 3.17 ± 1.92 | 4.05 ± 2.86 | 7.96 ± 2.15* | 3.38 ± 1.24^#^ |
| P15684 | 6.09 ± 1.29 | 5.39 ± 3.83 | 3.76 ± 8.16* | 6.70 ± 5.99 |
| P15800 | 2.14 ± 0.96 | 2.96 ± 9.50 | 0.89 ± 9.85* | 2.66 ± 1.57 |
| P16409 | 1.82 ± 7.71 | 4.01 ± 2.58* | 4.60 ± 4.72 | 5.55 ± 5.26^#^ |
| P16884 | 1.34 ± 2.85 | 1.45 ± 2.16 | 8.41 ± 1.12* | 5.08 ± 2.06 |
| P17077 | 1.01 ± 0.55 | 1.69 ± 7.64 | 0.70 ± 0.96 | 2.62 ± 1.02^#^ |
| P19527 | 1.22 ± 5.00 | 1.58 ± 2.38 | 4.06 ± 0.05* | 6.71 ± 2.57 |
| P20059 | 5.29 ± 2.72 | 3.08 ± 4.07 | 9.22 ± 6.34 | 1.87 ± 0.76^#^ |
| P20069 | 1.56 ± 8.06 | 3.46 ± 1.76* | 1.31 ± 1.68 | 2.45 ± 3.41 |
| P20070 | 6.78 ± 1.54 | 5.22 ± 1.95 | 5.14 ± 2.71* | 4.94 ± 1.45 |
| P20280 | 1.31 ± 7.42 | 1.46 ± 0.65 | 1.37 ± 4.55 | 6.67 ± 3.96^#^ |
| P20294 | 2.21 ± 1.09 | 4.46 ± 2.43* | 3.14 ± 1.75 | 2.99 ± 7.39 |
| P20760 | 2.90 ± 1.19 | 3.70 ± 5.64 | 2.03 ± 0.32 | 1.01 ± 1.35^#^ |
| P20961 | 7.95 ± 8.05 | 3.68 ± 2.97* | 6.85 ± 5.62 | 3.33 ± 3.94 |
| P21670 | 1.20 ± 1.90 | 3.17 ± 3.39 | 1.37 ± 0.37* | 2.88 ± 1.89^#^ |
| P23457 | 2.16 ± 8.40 | 2.65 ± 4.67 | 3.19 ± 1.30* | 1.57 ± 1.16 |
| P23514 | 7.43 ± 3.93 | 2.15 ± 2.34* | 9.66 ± 2.83 | 3.91 ± 4.77 |
| P24368 | 4.97 ± 1.67 | 4.76 ± 3.82 | 3.07 ± 3.58 | 9.30 ± 6.26^#^ |
| P25030 | 3.48 ± 2.64 | 4.83 ± 1.93 | 2.09 ± 2.82 | 7.10 ± 5.03^#^ |
| P29315 | 2.94 ± 1.71 | 4.70 ± 1.04* | 3.91 ± 2.83 | 7.00 ± 5.56 |
| P30904 | 1.41 ± 2.04 | 2.48 ± 3.86 | 1.47 ± 2.30 | NA |
| P31232 | 5.12 ± 3.49 | 1.72 ± 7.68* | 2.19 ± 3.02 | 2.14 ± 2.22 |
| P32362 | 5.50 ± 1.75 | 8.30 ± 1.21* | 4.89 ± 8.27 | 3.43 ± 3.27^#^ |
| P34058 | 4.67 ± 0.71 | 6.52 ± 1.74* | 4.09 ± 6.17 | 6.53 ± 5.58 |
| P35284 | 2.69 ± 1.54 | 2.52 ± 1.13 | 3.60 ± 1.29 | 1.71 ± 1.53^#^ |
| P35289 | 3.10 ± 3.51 | 2.69 ± 1.20* | 3.14 ± 1.20 | 1.83 ± 1.36^#^ |
| P35427 | 1.65 ± 0.98 | 2.23 ± 0.89 | 2.46 ± 5.04* | 4.01 ± 2.67 |
| P35465 | 5.28 ± 2.79 | 4.85 ± 3.04 | 0.69 ± 0.62* | 5.24 ± 2.71 |
| P35704 | 4.57 ± 3.26 | 5.19 ± 3.37 | 6.55 ± 5.57 | 2.41 ± 3.13^#^ |
| P38650 | 2.81 ± 1.38 | 3.32 ± 6.33* | 1.58 ± 1.84 | 3.09 ± 2.33 |
| P38652 | 5.03 ± 2.46 | 4.70 ± 3.35 | 3.67 ± 1.14 | 1.58 ± 1.24^#^ |
| P38918 | 4.32 ± 3.19 | 5.91 ± 1.58 | 7.90 ± 1.24 | 3.94 ± 3.48^#^ |
| P43245 | 2.63 ± 2.36 | 2.48 ± 2.35 | 8.75 ± 1.56* | 3.31 ± 9.81 |
| P45592 | 2.24 ± 2.70 | 3.20 ± 3.32 | 9.14 ± 0.76* | 6.57 ± 6.29 |
| Supplementar Table 1. Proteins Concentration Datasheet (Continuation) | | | |  |
| **Proteins** | **PC** | **PL** | **PW** | **PWL** |
|  | **Mean ± SD (x10^6)** | **Mean ± SD (x10^6)** | **Mean ± SD (x10^6)** | **Mean ± SD (x10^6)** |
| P47819 | 2.93 ± 0.90 | 2.09 ± 0.20* | 2.47 ± 0.39 | 5.97 ± 4.30 |
| P47875 | 4.02 ± 2.32 | 6.82 ± 1.82* | 5.11 ± 6.24 | 6.22 ± 4.12 |
| P47967 | 1.25 ± 6.81 | 2.58 ± 1.22* | 0.90 ± 1.03 | 4.33 ± 4.00 |
| P48004 | 2.41 ± 1.45 | 4.93 ± 2.49* | 2.06 ± 2.44 | 3.57 ± 2.73 |
| P48037 | 1.22 ± 6.85 | 1.58 ± 7.96 | 5.03 ± 5.70* | 4.13 ± 1.42 |
| P50116 | 1.15 ± 4.66 | 2.41 ± 2.14 | 2.40 ± 2.13* | 3.93 ± 3.63 |
| P50399 | 5.35 ± 1.04 | 4.11 ± 2.01 | 4.71 ± 1.05* | 6.04 ± 2.18 |
| P50503 | 2.30 ± 9.62 | 4.74 ± 1.57* | 1.47 ± 1.73 | 6.61 ± 5.88^#^ |
| P52296 | 4.37 ± 1.76 | 4.65 ± 2.60 | 2.63 ± 9.59* | 5.50 ± 9.55 |
| P52631 | 1.58 ± 1.08 | 5.58 ± 3.63* | 1.11 ± 0.39 | 3.09 ± 3.06 |
| P55159 | 6.69 ± 2.35 | 4.45 ± 3.61 | 5.58 ± 1.61 | 3.14 ± 1.70^#^ |
| P56574 | 6.54 ± 1.64 | 4.28 ± 3.42 | 5.49 ± 0.15* | 4.69 ± 1.06 |
| P58775 | 1.36 ± 5.58 | 2.01 ± 1.68* | 1.51 ± 1.79 | 1.74 ± 1.54 |
| P60123 | 2.25 ± 2.69 | 2.74 ± 2.84 | 1.12 ± 0.33 | 5.41 ± 5.91^#^ |
| P60901 | 3.55 ± 1.32 | 3.17 ± 2.47 | 3.65 ± 2.65 | 7.67 ± 4.28^#^ |
| P61107 | 3.92 ± 1.56 | 4.15 ± 1.57 | 1.50 ± 1.71* | 5.30 ± 2.81 |
| P61980 | 4.13 ± 2.33 | 5.66 ± 2.60 | 3.45 ± 3.93 | 0.70 ± 2.32^#^ |
| P62083 | 1.48 ± 1.29 | 2.48 ± 1.92 | 9.35 ± 4.02* | 2.74 ± 1.27 |
| P62198 | 5.51 ± 1.26 | 5.02 ± 3.55 | 3.14 ± 7.51* | 6.23 ± 5.73 |
| P62278 | 2.17 ± 1.80 | 3.40 ± 2.85 | 1.17 ± 1.59 | 4.72 ± 9.97^#^ |
| P62282 | 0.91 ± 6.09 | 1.66 ± 0.95 | 1.79 ± 1.29* | 3.16 ± 2.19 |
| P62628 | 2.14 ± 1.47 | 3.63 ± 1.18 | 2.46 ± 0.36 | 5.32 ± 3.86^#^ |
| P62703 | 0.76 ± 5.51 | 1.15 ± 5.39 | 1.24 ± 1.44* | 0.76 ± 1.07 |
| P62832 | 2.37 ± 8.56 | 3.62 ± 2.38 | 2.72 ± 4.32 | 1.13 ± 3.04^#^ |
| P62853 | 2.05 ± 1.94 | 2.69 ± 1.36 | 1.87 ± 7.70* | 2.55 ± 1.39 |
| P62898 | 2.50 ± 2.30 | 2.45 ± 1.63 | 0.97 ± 1.11 | 5.38 ± 5.62^#^ |
| P62912 | 4.55 ± 3.11 | 7.88 ± 1.12* | 1.10 ± 1.28* | 0.65 ± 4.87 |
| P63025 | 3.21 ± 2.07 | 1.98 ± 1.33 | 2.55 ± 5.69 | 0.43 ± 3.02^#^ |
| P63039 | 1.65 ± 0.69 | 2.60 ± 8.14* | 1.14 ± 1.52 | 1.87 ± 9.05 |
| P63259 | 1.35 ± 2.16 | 1.11 ± 5.07 | 1.08 ± 7.05* | 4.39 ± 9.66 |
| P63269 | 2.30 ± 2.98 | 2.16 ± 2.10 | 9.06 ± 7.24* | 3.06 ± 8.74 |
| P67779 | 2.65 ± 2.50 | 1.64 ± 9.30 | 6.65 ± 2.43* | 3.01 ± 2.29 |
| P68370 | 2.20 ± 0.14 | 1.49 ± 7.19* | 1.98 ± 7.11* | 1.45 ± 1.10 |
| P68511 | 3.84 ± 1.49 | 5.55 ± 1.13* | 5.13 ± 2.31 | 2.42 ± 4.20^#^ |
| P69897 | 4.15 ± 3.60 | 3.08 ± 1.57 | 3.52 ± 0.21* | 2.61 ± 1.91 |
| P70490 | 6.89 ± 2.80 | 1.13 ± 7.37* | 1.40 ± 1.70* | 4.27 ± 3.53 |
| P70580 | 3.83 ± 2.55 | 6.62 ± 2.05* | 7.34 ± 2.23 | 4.06 ± 7.22 |
| P70615 | 2.37 ± 2.59 | 3.73 ± 3.76 | 7.80 ± 1.05* | 5.56 ± 5.10^#^ |
| P70617 | 3.65 ± 1.88 | 1.25 ± 1.36* | 4.02 ± 1.30 | 1.85 ± 8.92 |
| P82471 | 2.55 ± 7.53 | 3.22 ± 2.64 | 2.66 ± 3.55 | 6.59 ± 4.12^#^ |
| P82995 | 4.34 ± 5.56 | 5.72 ± 1.24* | 3.43 ± 3.70 | 5.94 ± 5.47 |
| P83732 | 1.97 ± 1.65 | 1.51 ± 8.00 | 8.73 ± 1.60* | 0.83 ± 0.12 |
| P84083 | 2.26 ± 2.68 | 1.50 ± 2.25 | 7.72 ± 1.24* | 6.57 ± 3.21 |
| P84092 | 7.68 ± 6.99 | 4.85 ± 4.21 | 5.19 ± 0.57* | 0.89 ± 9.32 |
| P85515 | 5.83 ± 1.50 | 2.89 ± 2.06* | 2.70 ± 3.15 | 1.63 ± 8.28 |
| Supplementar Table 1. Proteins Concentration Datasheet (Continuation) | | | |  |
| **Proteins** | **PC** | **PL** | **PW** | **PWL** |
|  | **Mean ± SD (x10^6)** | **Mean ± SD (x10^6)** | **Mean ± SD (x10^6)** | **Mean ± SD (x10^6)** |
| P85968 | 3.74 ± 1.41 | 4.48 ± 1.90 | 3.31 ± 3.25 | 6.04 ± 3.79^#^ |
| P85971 | 6.60 ± 2.18 | 5.99 ± 2.30 | 7.77 ± 8.39 | 2.47 ± 9.40^#^ |
| Q00438 | 2.95 ± 5.86 | 2.42 ± 1.63 | 1.07 ± 1.26* | 5.01 ± 6.28 |
| Q02874 | 1.86 ± 8.32 | 3.29 ± 2.94 | 8.29 ± 2.04* | 4.53 ± 4.26 |
| Q05982 | 7.12 ± 3.45 | 2.27 ± 3.42* | 9.38 ± 6.04 | 4.66 ± 6.48 |
| Q08163 | 3.93 ± 3.97 | 1.53 ± 9.12 | 1.05 ± 4.18 | 4.33 ± 2.63^#^ |
| Q2KJ09 | 1.53 ± 1.24 | 3.13 ± 2.91 | 3.81 ± 3.99* | 5.70 ± 3.25 |
| Q2PQA9 | 7.31 ± 2.77 | 7.66 ± 1.92 | 8.58 ± 7.92 | 4.19 ± 3.09^#^ |
| Q3KRD5 | 6.04 ± 2.42 | 2.70 ± 2.55* | 5.55 ± 1.28 | 2.62 ± 2.92 |
| Q3KRE8 | 2.04 ± 1.65 | 1.70 ± 3.92* | 2.04 ± 2.86 | 1.49 ± 9.93^#^ |
| Q3T1I4 | 5.51 ± 4.17 | 4.39 ± 3.00 | 2.68 ± 3.04* | 3.63 ± 1.76 |
| Q4FZT0 | 2.01 ± 2.25 | 6.15 ± 4.02* | 1.02 ± 3.73 | 3.37 ± 1.11 |
| Q4KLM6 | 1.69 ± 4.13 | 1.75 ± 1.20 | 1.14 ± 0.43* | 2.08 ± 2.12 |
| Q4V7C7 | 2.49 ± 1.76 | 3.31 ± 1.46 | 3.46 ± 0.11 | 2.72 ± 1.77^#^ |
| Q4V8F9 | 4.41 ± 3.15 | 3.04 ± 3.48 | 4.30 ± 1.40 | 0.75 ± 2.21^#^ |
| Q561Q8 | 3.94 ± 1.23 | 3.61 ± 2.54 | 2.85 ± 6.79* | 2.16 ± 1.16 |
| Q5BJP3 | 1.63 ± 1.31 | 1.98 ± 1.28 | 3.82 ± 4.69* | 2.31 ± 2.42 |
| Q5BJY9 | 6.11 ± 3.36 | 6.22 ± 3.02 | 6.59 ± 6.32 | 1.96 ± 3.69^#^ |
| Q5HZY2 | 6.00 ± 4.18 | 0.92 ± 6.12* | 5.41 ± 6.15 | 4.70 ± 9.64 |
| Q5PQK1 | 7.16 ± 2.54 | 2.65 ± 2.26* | 4.18 ± 5.76 | 5.89 ± 4.71 |
| Q5RJP9 | 2.33 ± 1.58 | 2.10 ± 1.79 | 4.80 ± 3.72 | 0.91 ± 1.28^#^ |
| Q5RK30 | 3.49 ± 1.68 | 3.25 ± 1.75 | 2.23 ± 2.52* | 2.23 ± 2.97 |
| Q5RKL5 | 1.71 ± 1.29 | 2.27 ± 1.20 | 2.51 ± 4.44* | 1.93 ± 2.58 |
| Q5U211 | 2.05 ± 4.19 | 1.45 ± 7.45 | 1.86 ± 1.50 | 7.23 ± 5.53^#^ |
| Q5U2Q7 | 2.37 ± 3.72 | 1.55 ± 1.05 | 2.02 ± 7.16* | 2.01 ± 1.83 |
| Q5U2X6 | 1.45 ± 6.43 | 2.45 ± 1.72 | 7.99 ± 2.94* | 1.57 ± 1.38 |
| Q5U316 | 3.30 ± 3.56 | 2.57 ± 1.14 | 3.30 ± 5.18 | 2.44 ± 1.53^#^ |
| Q5XI72 | 1.32 ± 1.45 | 2.86 ± 3.06 | 8.68 ± 1.76* | 1.38 ± 1.10 |
| Q5XIF6 | 1.88 ± 2.36 | 1.69 ± 3.34 | 1.68 ± 2.22* | 1.05 ± 7.78 |
| Q5XIM5 | 4.18 ± 2.42 | 6.43 ± 2.01 | 6.79 ± 3.00* | 6.22 ± 4.85 |
| Q5XXR3 | 2.28 ± 2.70 | 1.38 ± 2.94 | 8.45 ± 5.35* | 5.75 ± 1.20 |
| Q62871 | 3.01 ± 3.89 | 2.20 ± 1.84 | 9.34 ± 4.16* | 4.02 ± 2.16 |
| Q62925 | 2.92 ± 1.55 | 2.94 ± 1.44 | 3.55 ± 8.72 | 1.18 ± 8.12^#^ |
| Q63016 | 4.28 ± 3.23 | 1.11 ± 8.43* | 1.10 ± 1.24 | 2.57 ± 9.85 |
| Q63081 | 3.62 ± 2.24 | 4.38 ± 1.74 | 2.26 ± 1.56 | 4.06 ± 2.26^#^ |
| Q63088 | 1.80 ± 7.49 | 2.94 ± 1.05* | 1.74 ± 2.23 | 4.61 ± 3.50 |
| Q63355 | 4.86 ± 8.88 | 3.15 ± 2.52 | 3.55 ± 5.44* | 2.28 ± 2.02 |
| Q63356 | 3.11 ± 3.58 | 3.53 ± 3.46 | 6.47 ± 6.04* | 2.24 ± 4.67^#^ |
| Q63377 | 5.31 ± 3.73 | 7.41 ± 1.29 | 7.94 ± 1.49 | 3.64 ± 9.73^#^ |
| Q63525 | 1.34 ± 5.69 | 2.91 ± 2.71 | 8.87 ± 1.34* | 6.02 ± 4.85 |
| Q63610 | 7.59 ± 1.16 | 7.39 ± 1.40 | 3.45 ± 3.96* | 2.56 ± 3.29 |
| Q63678 | 2.04 ± 1.39 | 3.15 ± 3.01 | 8.14 ± 4.61* | 5.86 ± 3.43 |
| Q63716 | 2.21 ± 3.08 | 2.48 ± 3.30 | 7.43 ± 8.88* | 4.57 ± 8.48 |
| Q63797 | 3.09 ± 1.43 | 2.75 ± 1.36 | 3.13 ± 3.19 | 1.83 ± 5.45^#^ |
|  |  |  |  |  |
| Supplementar Table 1. Proteins Concentration Datasheet (Continuation) | | | |  |
| **Proteins** | **PC** | **PL** | **PW** | **PWL** |
|  | **Mean ± SD (x10^6)** | **Mean ± SD (x10^6)** | **Mean ± SD (x10^6)** | **Mean ± SD (x10^6)** |
| Q63965 | 6.29 ± 2.85 | 6.00 ± 4.00 | 3.87 ± 1.21* | 4.84 ± 1.40 |
| Q641X8 | 1.19 ± 6.86 | 2.00 ± 7.20 | 1.78 ± 9.32 | 2.79 ± 3.12^#^ |
| Q641Y0 | 3.50 ± 4.08 | 2.93 ± 1.29 | 2.57 ± 1.65* | 2.17 ± 2.88 |
| Q64303 | 2.91 ± 2.62 | 3.91 ± 3.39 | 1.34 ± 3.62 | 9.50 ± 6.86^#^ |
| Q64428 | 1.70 ± 9.94 | 2.64 ± 2.74 | 1.07 ± 6.55 | 9.27 ± 6.92^#^ |
| Q64559 | 3.71 ± 2.03 | 4.75 ± 2.52 | 1.34 ± 2.61* | 1.11 ± 0.00 |
| Q66H61 | 2.05 ± 0.30 | 1.99 ± 6.19 | 0.70 ± 7.51* | 1.65 ± 8.58 |
| Q66HA1 | 2.97 ± 3.01 | 1.59 ± 0.37 | 8.59 ± 0.78* | 5.64 ± 3.48 |
| Q66HF1 | 4.08 ± 1.99 | 4.04 ± 1.12 | 4.31 ± 3.02 | 3.46 ± 3.88^#^ |
| Q66X93 | 6.84 ± 1.55 | 6.44 ± 2.77 | 3.96 ± 8.14* | 2.75 ± 7.20 |
| Q68A21 | 3.01 ± 3.22 | 2.01 ± 2.22 | 0.61 ± 6.60 | 7.35 ± 6.09^#^ |
| Q68FP1 | 5.99 ± 4.19 | 6.34 ± 4.10 | 8.52 ± 9.06 | 0.65 ± 4.55^#^ |
| Q68FQ0 | 3.43 ± 7.03 | 3.71 ± 1.21 | 2.29 ± 2.85* | 1.67 ± 1.26 |
| Q68FR8 | 1.99 ± 1.32 | 1.36 ± 6.83* | 1.79 ± 3.26* | 1.22 ± 9.37 |
| Q68FY0 | 7.77 ± 3.04 | 2.98 ± 3.37* | 8.19 ± 1.96 | 2.98 ± 8.08 |
| Q6AY09 | 7.97 ± 2.35 | 3.64 ± 4.37* | 1.02 ± 0.58* | 1.30 ± 0.89 |
| Q6AY22 | 3.30 ± 1.95 | 4.34 ± 1.53 | 2.50 ± 1.78 | 4.92 ± 3.06^#^ |
| Q6IE52 | 4.23 ± 4.68 | 3.77 ± 1.82 | 2.99 ± 3.54* | 3.38 ± 2.85 |
| Q6IFU8 | 6.99 ± 3.78 | 1.67 ± 1.15* | 7.08 ± 5.76 | 3.53 ± 2.19 |
| Q6IFV1 | 6.64 ± 3.66 | 1.80 ± 9.29* | 6.71 ± 9.51 | 3.38 ± 2.20 |
| Q6IFV4 | 2.02 ± 3.21 | 1.72 ± 1.17 | 8.86 ± 5.65* | 1.85 ± 2.54 |
| Q6IFW2 | 4.87 ± 1.71 | 4.39 ± 2.40 | 1.23 ± 1.36* | 2.75 ± 2.03 |
| Q6IFW5 | 5.17 ± 6.00 | 6.37 ± 7.12* | 3.93 ± 7.69 | 2.65 ± 5.77 |
| Q6IG00 | 2.24 ± 2.52 | 1.79 ± 8.94 | 0.77 ± 8.51* | 5.66 ± 1.45 |
| Q6IG04 | 2.79 ± 3.33 | 4.91 ± 4.25 | 5.27 ± 5.97* | 6.11 ± 7.02 |
| Q6IG05 | 2.64 ± 1.13 | 4.16 ± 1.28* | 2.38 ± 2.72 | 3.34 ± 2.84 |
| Q6P502 | 7.97 ± 1.36 | 7.09 ± 2.94 | 7.03 ± 6.55* | 7.03 ± 5.97 |
| Q6P6S0 | 3.61 ± 1.62 | 5.83 ± 1.74* | 2.14 ± 4.52 | 3.66 ± 8.28 |
| Q6P747 | 4.83 ± 1.01 | 5.18 ± 2.06 | 5.11 ± 4.72 | 7.18 ± 5.19^#^ |
| Q6P7B0 | 2.87 ± 0.66 | 2.77 ± 9.48 | 2.23 ± 7.19* | 0.91 ± 1.28 |
| Q6P9T8 | 3.62 ± 2.56 | 3.27 ± 7.56 | 2.98 ± 2.34* | 1.38 ± 1.79^#^ |
| Q6Q7Y5 | 6.88 ± 1.06 | 2.68 ± 3.41* | 7.31 ± 2.10 | 6.74 ± 2.94 |
| Q7M0E3 | 2.97 ± 2.52 | 2.42 ± 9.10 | 2.24 ± 2.15 | 1.55 ± 8.58^#^ |
| Q7TP47 | 4.93 ± 1.79 | 5.31 ± 2.80 | 4.78 ± 0.11 | 2.25 ± 1.88^#^ |
| Q7TPB1 | 8.03 ± 1.24 | 5.46 ± 3.71 | 3.69 ± 5.11* | 4.93 ± 4.56 |
| Q7TPJ0 | 6.39 ± 2.45 | 5.35 ± 2.08 | 5.67 ± 2.52 | 1.90 ± 9.75^#^ |
| Q7TQ94 | 1.45 ± 5.14 | 2.01 ± 3.74* | 1.53 ± 1.87 | 5.98 ± 1.73^#^ |
| Q8R491 | 4.09 ± 3.75 | 3.27 ± 1.94 | 3.62 ± 2.30* | 3.57 ± 1.56 |
| Q8VHF5 | 3.51 ± 0.70 | 3.28 ± 1.45 | 1.39 ± 1.68* | 7.05 ± 6.62 |
| Q8VIF7 | 5.60 ± 3.22 | 3.34 ± 3.13 | 2.39 ± 9.56* | 6.02 ± 6.38 |
| Q920P6 | 6.22 ± 2.81 | 4.17 ± 3.42 | 4.58 ± 9.13 | 3.99 ± 3.25^#^ |
| Q99376 | 1.71 ± 2.86 | 1.36 ± 6.20 | 1.31 ± 0.32* | 1.38 ± 1.24 |
| Q99MB4 | 1.74 ± 0.50 | 2.45 ± 2.82 | 4.07 ± 4.06* | 0.97 ± 0.81 |
| Q99MZ8 | 4.81 ± 1.72 | 4.52 ± 1.99 | 2.96 ± 2.25* | 4.71 ± 3.96 |
| Q63862 | 1.92 ± 5.21 | 1.74 ± 8.83 | 1.55 ± 6.04 | 3.51 ± 2.65^#^ |
| Supplementar Table 1. Proteins Concentration Datasheet (Continuation) | | | |  |
| **Proteins** | **PC** | **PL** | **PW** | **PWL** |
|  | **Mean ± SD (x10^6)** | **Mean ± SD (x10^6)** | **Mean ± SD (x10^6)** | **Mean ± SD (x10^6)** |
| Q99PF5 | 6.11 ± 3.20 | 1.08 ± 2.85* | 5.63 ± 4.96 | 2.68 ± 7.20 |
| Q9EPH2 | 1.54 ± 1.19 | 1.49 ± 2.89 | 1.63 ± 2.04 | 0.89 ± 1.17^#^ |
| Q9ER24 | 2.61 ± 3.97 | 3.29 ± 2.56 | 0.87 ± 1.02* | 3.48 ± 1.41^#^ |
| Q9ESN0 | 4.25 ± 2.35 | 3.01 ± 1.83 | 4.48 ± 1.39 | 1.69 ± 1.11^#^ |
| Q9EST6 | 5.80 ± 3.11 | 4.12 ± 3.04 | 5.56 ± 2.19 | 3.32 ± 2.65^#^ |
| Q9QUL6 | 4.57 ± 1.05 | 4.23 ± 2.06 | 4.20 ± 6.47* | 6.88 ± 3.76 |
| Q9QX79 | 1.76 ± 9.61 | 2.68 ± 4.48 | 2.56 ± 1.18* | 2.12 ± 1.08 |
| Q9QY17 | 5.30 ± 2.41 | 8.02 ± 1.80* | 5.80 ± 3.26 | 4.51 ± 2.25 |
| Q9QZM5 | 8.19 ± 2.78 | 2.42 ± 3.23* | 4.50 ± 5.03 | 4.59 ± 3.99 |
| Q9R006 | 2.88 ± 6.99 | 2.52 ± 1.58 | 1.13 ± 1.39* | 0.67 ± 8.76 |
| Q9R063 | 2.96 ± 1.24 | 4.31 ± 4.40* | 2.36 ± 3.42 | 6.06 ± 2.82 |
| Q9WVA9 | 2.69 ± 0.92 | 2.62 ± 6.08 | 0.91 ± 9.72 | 2.86 ± 1.86^#^ |
| Q9WVK7 | 6.45 ± 2.23 | 4.09 ± 3.69 | 5.35 ± 1.39 | 3.03 ± 2.49^#^ |
| Q9Z0V5 | 3.34 ± 4.10 | 3.53 ± 1.63 | 1.05 ± 1.16* | 4.09 ± 2.34 |
| Q9Z1A6 | 2.11 ± 1.29 | 3.32 ± 4.57* | 2.38 ± 1.45 | 2.24 ± 2.29 |
| Q9Z270 | 6.25 ± 3.04 | 5.70 ± 3.80 | 3.81 ± 0.86* | 2.20 ± 1.20 |
| Q9Z2L0 | 2.61 ± 2.60 | 1.43 ± 0.79 | 9.47 ± 7.18* | 4.26 ± 4.89 |

Suplementar Table 1. Protein concentrations from proteomics analysis. Concentrations are presented in average + SD for each group. Legend: PC: control pregnant group; PL: pregnant rats fed a 3% leucine-rich diet; PW: pregnant Walker tumour-bearing group; PWL: tumour-bearing pregnant rats fed a 3% leucine-rich diet. Values are presented in means $\pm$ standard deviation (SD). * p < 0.05 difference against PC group; ** p < 0.05 difference against PL group. Values obtained via two-way ANOVA analysis followed by Tukey’s post-test

| Supplementar Table 2. Metabolites Concentrations Datasheet | | | | | | |  |  |  |  |  |  |
| --- | --- | --- | --- | --- | --- | --- | --- | --- | --- | --- | --- | --- |
| **Metabolites** | **PC** | | | **PL** | | | **PW** | | | **PWL** | | |
|  | **Mean ± SD** | | | **Mean ± SD** | | | **Mean ± SD** | | | **Mean ± SD** | | |
| 2-aminobutyrate | 100.0 | ± | 17.4 | 99.4 | ± | 66.6 | 83.0 | ± | 41.2 | 106.3 | ± | 34.3 |
| 2-Phosphoglycerate | 100.0 | ± | 32.2 | 149.3 | ± | 42.5 | 88.9 | ± | 57.4 | 197.1 | ± | 65.4 |
| 3-Hydroxyisobutyrate | 100.0 | ± | 19.8 | 142.1 | ± | 33.0 | 127.5 | ± | 42.9 | 124.4 | ± | 20.2 |
| 3-Hydroxyisovalerate | 100.0 | ± | 74.1 | 52.1 | ± | 23.5 | 92.2 | ± | 61.6 | 74.1 | ± | 28.2 |
| acetate | 100.0 | ± | 19.0 | 73.7 | ± | 10.9 | 131.2 | ± | 55.8 | 142.9 | ± | 35.5^#^ |
| adenine | 100.0 | ± | 8.2 | 542.2 | ± | 4.5 | 83.2 | ± | 26.2* | 78.3 | ± | 3.5^#^ |
| adenosine | 100.0 | ± | 65.6 | 62.9 | ± | 28.6 | 33.0 | ± | 9.5* | 101.7 | ± | 53.4 |
| aDP | 100.0 | ± | 77.8 | 519.2 | ± | 142.4* | 133.1 | ± | 85.2 | 137.3 | ± | 34.8^#^ |
| alanine | 100.0 | ± | 16.3 | 94.7 | ± | 8.1 | 156.7 | ± | 58.7 | 115.1 | ± | 11.5 |
| aMP | 100.0 | ± | 46.1 | 995.1 | ± | 363.3* | 129.1 | ± | 75.4 | 278.5 | ± | 151.1^#^ |
| asparagine | 100.0 | ± | 12.8 | 69.8 | ± | 9.6* | 84.8 | ± | 12.7 | 133.4 | ± | 24.1^#^ |
| aspartate | 100.0 | ± | 12.2 | 110.2 | ± | 25.3 | 68.1 | ± | 18.5* | 113.6 | ± | 20.3 |
| aTP | 100.0 | ± | 32.0 | 208.0 | ± | 46.0* | 96.0 | ± | 51.3 | 168.2 | ± | 90.1 |
| betaine | 100.0 | ± | 18.4 | 151.4 | ± | 31.2 | 60.0 | ± | 52.0 | 62.3 | ± | 10.4^#^ |
| carnitine | 100.0 | ± | 7.7 | 121.9 | ± | 35.0 | 133.8 | ± | 128.8 | 83.0 | ± | 12.2 |
| choline | 100.0 | ± | 16.4 | 81.7 | ± | 15.9 | 75.2 | ± | 39.0 | 99.0 | ± | 6.6 |
| citrate | 100.0 | ± | 18.5 | 92.0 | ± | 59.5 | 75.9 | ± | 20.3 | 106.5 | ± | 31.6 |
| creatine | 100.0 | ± | 24.3 | 101.8 | ± | 14.1 | 192.1 | ± | 34.4* | 102.3 | ± | 41 |
| creatine phosphate | 100.0 | ± | 16.5 | 103.2 | ± | 18.3 | 131.4 | ± | 31.8 | 142.8 | ± | 30.3 |
| cytidine | 100.0 | ± | 43.5 | 109.0 | ± | 35.7 | 143.4 | ± | 54.4 | 145.5 | ± | 64.9 |
| Ethanolamine | 100.0 | ± | 5.7 | 753.1 | ± | 5.1* | 96.8 | ± | 12.4 | 116.0 | ± | 12.9^#^ |
| Formate | 100.0 | ± | 91.9 | 74.3 | ± | 25.3 | 102.6 | ± | 67.1 | 101.5 | ± | 33.8 |
| Fumarate | 100.0 | ± | 15.2 | 72.46 | ± | 18.2 | 118.5 | ± | 9.7 | 114.5 | ± | 33.8^#^ |
| Gluconate | 100.0 | ± | 32.6 | 78.5 | ± | 33.1 | 72.3 | ± | 37.3 | 106.4 | ± | 21.7 |
| Glucose | 100.0 | ± | 23.2 | 27.1 | ± | 8.3* | 63.5 | ± | 19.4* | 59.7 | ± | 11.2^#^ |
| Glucose-6-phosphate | 100.0 | ± | 46.9 | 29.8 | ± | 18.1* | 57.4 | ± | 22.2 | 93.4 | ± | 29.0^#^ |
| Glutamate | 100.0 | ± | 10.8 | 105.3 | ± | 14.7 | 70.2 | ± | 16.7* | 91.8 | ± | 15.7 |
| Glutamine | 100.0 | ± | 30.3 | 91.3 | ± | 21.8 | 92.7 | ± | 24.9 | 97.4 | ± | 6.5 |
| Glutathione | 100.0 | ± | 28.8 | 179.8 | ± | 33.9* | 44.4 | ± | 43.0 | 114.0 | ± | 66.6^#^ |
| Glycine | 100.0 | ± | 16.2 | 64.0 | ± | 14.4* | 101.7 | ± | 19.5 | 109.2 | ± | 15.4^#^ |
| GTP | 100.0 | ± | 40.0 | 166.1 | ± | 35.2 | 122.0 | ± | 34.7 | 199.4 | ± | 87.3 |
| Guanidoacetate | 100.0 | ± | 62.4 | 96.0 | ± | 56.7 | 71.3 | ± | 54.2 | 134.2 | ± | 13.3 |
| Guanosine | 100.0 | ± | 36.2 | 549.0 | ± | 70.5* | 85.0 | ± | 24.4 | 166.0 | ± | 108.4^#^ |
| Histamine | 100.0 | ± | 40.9 | 97.2 | ± | 28.3 | 176.8 | ± | 32.2* | 187.7 | ± | 64 |
| Histidine | 100.0 | ± | 21.0 | 74.7 | ± | 22.3 | 93.2 | ± | 63.0 | 128.7 | ± | 35.8 |
| IMP | 100.0 | ± | 65.8 | 174.2 | ± | 26.2 | 64.1 | ± | 48.1 | 69.4 | ± | 42.8^#^ |
| Inosine | 100.0 | ± | 22.3 | 179.8 | ± | 13.7* | 77.6 | ± | 30.0 | 101.4 | ± | 20.4^#^ |
| Isoleucine | 100.0 | ± | 9.4 | 61.0 | ± | 11.0* | 94.4 | ± | 8.2 | 112.1 | ± | 15.4^#^ |
| Lactate | 100.0 | ± | 8.5 | 115.2 | ± | 7.1 | 126.6 | ± | 19.1* | 96.0 | ± | 11.7^#^ |
| Leucine | 100.0 | ± | 6.5 | 91.0 | ± | 25.7 | 95.0 | ± | 8.7 | 128.9 | ± | 28.6^#^ |
| Lysine | 100.0 | ± | 13.2 | 104.0 | ± | 9.2 | 103.8 | ± | 17.2 | 123.3 | ± | 10.8 |
| Methionine | 100.0 | ± | 13.1 | 57.6 | ± | 8.0* | 87.9 | ± | 8.9 | 109.7 | ± | 20.3^#^ |
| myo-Inositol | 100.0 | ± | 12.3 | 97.7 | ± | 15.5 | 120.4 | ± | 32.3 | 140.1 | ± | 43.0 |
| NaD+ | 100.0 | ± | 84.9 | 383.9 | ± | 91.8* | 39.7 | ± | 22.9 | 151.7 | ± | 99.4^#^ |
| Nicotinurate | 100.0 | ± | 17.4 | 80.9 | ± | 12.9 | 86.0 | ± | 13.5 | 106.9 | ± | 23.8 |
| O-Phosphocholine | 100.0 | ± | 22.6 | 182.4 | ± | 23.3* | 76.8 | ± | 22.0 | 88.7 | ± | 15.0^#^ |
| O-Phosphoethanolamine | 100.0 | ± | 28.0 | 161.8 | ± | 21.4* | 121.1 | ± | 43.1 | 137.4 | ± | 10.3 |
| Pantothenate | 100.0 | ± | 38.4 | 63.8 | ± | 13.8* | 45.1 | ± | 10.2 | 68.2 | ± | 24.6 |
| Phenylalanine | 100.0 | ± | 16.0 | 65.7 | ± | 7.5* | 89.3 | ± | 20.4 | 113.3 | ± | 14.8^#^ |
| Proline | 100.0 | ± | 13.7 | 110.0 | ± | 23.1 | 107.6 | ± | 39.2 | 130.8 | ± | 22.6 |
| Pyridoxine | 100.0 | ± | 48.4 | 88.3 | ± | 34.8 | 42.8 | ± | 29.8 | 102.4 | ± | 60.2 |
| Pyruvate | 100.0 | ± | 75.2 | 82.3 | ± | 33.3 | 104.3 | ± | 46.3 | 135.5 | ± | 45.9 |
| Serine | 100.0 | ± | 11.6 | 63.3 | ± | 13.4* | 91.8 | ± | 19.5 | 111.9 | ± | 22.4^#^ |
| sn-Glycero-3-phosphocholine | 100.0 | ± | 17.5 | 343.3 | ± | 86.8* | 106.7 | ± | 21.6 | 107.8 | ± | 37.7^#^ |
| Succinate | 100.0 | ± | 19.6 | 202.4 | ± | 21.6* | 64.8 | ± | 20.9* | 96.9 | ± | 12.7^#^ |
| Taurine | 100.0 | ± | 18.4 | 99.3 | ± | 7.5 | 97.4 | ± | 37.8 | 98.7 | ± | 17.8 |
| Threonine | 100.0 | ± | 14.3 | 89.1 | ± | 10.1 | 91.1 | ± | 12.5 | 114.2 | ± | 20.7^#^ |
| Tryptophan | 100.0 | ± | 13.1 | 76.1 | ± | 7.7 | 88.2 | ± | 16.6 | 131.8 | ± | 26.9^#^ |
| Tyrosine | 100.0 | ± | 14.9 | 63.4 | ± | 8.7* | 103.0 | ± | 19.6 | 107.4 | ± | 17.6^#^ |
| UDP-glucose | 100.0 | ± | 46.4 | 902.8 | ± | 210.9* | 154.3 | ± | 51.6 | 173.3 | ± | 57.8^#^ |
| UDP-glucuronate | 100.0 | ± | 41.6 | 660.9 | ± | 180.7* | 141.7 | ± | 32.9 | 220.7 | ± | 83.4^#^ |
| UMP | 100.0 | ± | 33.7 | 625.3 | ± | 218.2* | 121.4 | ± | 35.3 | 197.1 | ± | 73.6^#^ |
| Uracil | 100.0 | ± | 12.3 | 34.7 | ± | 7.6* | 89.0 | ± | 13.4 | 77.9 | ± | 17.6^#^ |
| Uridine | 100.0 | ± | 40.5 | 126.7 | ± | 21.0 | 94.3 | ± | 46.7 | 163.1 | ± | 56.7 |
| Valine | 100.0 | ± | 8.6 | 67.5 | ± | 9.5* | 97.1 | ± | 10.1 | 108.2 | ± | 17.4^#^ |
| Xanthine | 100.0 | ± | 41.3 | 40.4 | ± | 15.2 | 123.9 | ± | 27.7 | 124.6 | ± | 52.9^#^ |
| β-alanine | 100.0 | ± | 27.0 | 133.9 | ± | 58.5 | 104.0 | ± | 22.2 | 128.7 | ± | 34.6 |
| π-Methylhistidine | 100.0 | ± | 29.6 | 87.0 | ± | 16.8 | 127.0 | ± | 30.2 | 117.9 | ± | 23.8 |

Supplementar Table 2.: Metabolites concentrations from 1H-NMR metabolomics. Data are presented as a percentage of PC (100%). Legend: PC: control pregnant group; PL: pregnant rats fed a 3% leucine-rich diet; PW: pregnant Walker tumour-bearing group; PWL: tumour-bearing pregnant rats fed a 3% leucine-rich diet. Values are presented in means ± standard deviation (SD). * p < 0.05 difference against PC group; ** p < 0.05 difference against PL group. Values obtained via two-way ANOVA analysis followed by Tukey’s post-test.
